# Synergistic anticancer effects of cisplatin and phenolic aglycones of the aerial part of *Rumex dentatus* L. in tongue squamous cell carcinoma: insights from network pharmacology and biological verification

**DOI:** 10.1186/s12906-024-04718-5

**Published:** 2025-01-25

**Authors:** Amany E. Ragab, Ghada M. Al-Ashmawy, Sherin R. El Afify, Ola A. El-Feky, Amera O. Ibrahim

**Affiliations:** 1 Department of Pharmacognosy, Faculty of Pharmacy, Tanta University, Tanta, 31527 Egypt; 2 Department of Biochemistry, Faculty of Pharmacy, Tanta University, Tanta, 31527 Egypt; 3 Department of Biochemistry, Faculty of Pharmacy, Alsalam University, Kafr Alzayat, Algharbia 31611 Egypt; 4 Department of Pharmacology and Toxicology, Faculty of Pharmacy, Alsalam University, Kafr Alzayat, Algharbia 31611 Egypt

**Keywords:** Oral Squamous Cell Carcinoma (OSCC), *Rumex dentatus* L., Cisplatin Resistance, Apoptosis, Cell Cycle Arrest, Network Pharmacology

## Abstract

**Background:**

Oral squamous cell carcinoma (OSCC) ranks as the sixth most common malignancy globally. Cisplatin is the standard chemotherapy for OSCC, but resistance often reduces its efficacy, necessitating new treatments with fewer side effects. *Rumex dentatus L*., from the Polygonaceae family, is known for its medicinal properties, but its anticancer potential has not been thoroughly explored. This study aimed to investigate the synergy between cisplatin and an extract from the aerial parts of *R. dentatus L*. in treating tongue carcinoma (HNO97) in vitro, using network pharmacology, biological verification, and phytochemical analysis.

**Methods:**

The study included UPLC-ESI–MS/MS analysis, cytotoxicity assays, cell cycle analysis, apoptosis assessment, and RT-qPCR for gene expression of Bcl2, p53, and ATG7. Potential targets were identified, and mechanisms of action were examined through online databases and enrichment analyses.

**Results:**

The *R. dentatus L*. extract contained 14 phenolic aglycons. Combining cisplatin and *R. dentatus L*. was more effective in inhibiting proliferation, inducing cell cycle arrest and apoptosis, and reducing autophagy in HNO97 cells than cisplatin alone. KEGG analysis indicated that the drug combination might work through pathways like PI3K-Akt signaling, microRNAs in cancer, and EGFR tyrosine kinase inhibitor resistance.

**Conclusions:**

Combining cisplatin with *R. dentatus L*. may be a promising approach for treating tongue carcinoma by affecting multiple pathways, providing a new perspective for developing more effective treatments for OSCC.

## Introduction

Oral cancer ranks as the sixth most prevalent malignancy globally [1]. The majority of oral malignancies manifest as oral squamous cell carcinoma (OSCC), with the tongue being the most frequently affected site. Patients with oral cancer encounter significant morbidity and mortality rates, coupled with poor survival outcomes, even post-surgery. Late diagnosis, early metastasis upon diagnosis, and local recurrence contribute to these challenges [2].

Cisplatin stands as the gold standard chemotherapeutic agent for OSCC treatment. By forming intra-strand adducts within genomic DNA, cisplatin directly damages DNA and disrupts its replication, arresting the cell cycle and ultimately inducing cell death [3]. Treatment with cisplatin typically leads to tumour shrinkage, facilitating the feasibility of radiotherapy and reducing surgery-associated disfigurement [4]. Despite the initial high response to cisplatin, the emergence of chemoresistance significantly curtails its efficacy, leading to treatment failure and cancer relapse [4]. Hence, there is an urgent need to develop new therapeutic strategies or chemopreventive alternatives with minimal toxicity to normal tissues to overcome cisplatin resistance, enhance its efficacy, and improve clinical outcomes in OSCC.

Apoptosis plays a pivotal role in carcinogenesis and treatment response across various tumors. Dysregulated anti-apoptotic proteins like BCl2 and reduced expression of pro-apoptotic proteins such as p53 are critical for cancer development and chemoresistance. Moreover, resistance to apoptosis correlates with chemoresistance [5]. A close interplay exists between apoptosis and autophagy, sharing common core mediators, upstream regulators, and signaling pathways. Emerging evidence suggests that autophagy significantly influences OSCC progression and chemoresistance regulation [6]. Studies have shown that the downregulation of autophagic genes like ATG5 or ATG7 inhibits tumour growth and enhances apoptosis, underscoring the indispensable role of autophagy in sustaining cancer cell growth [7, 8].

Previous findings have indicated that autophagy also contributes to chemotherapy resistance and cancer stemness. Cisplatin-resistant cell lines exhibit elevated levels of stemness markers and increased autophagic flux compared to parental OSCC cells. Inhibition of autophagy leads to reduced stemness [9]. Additionally, the pro-apoptotic protein p53 suppresses oncogenic potential by acting as a transcription factor that regulates the cell cycle, triggers apoptotic cell death, and modulates autophagy [10].

Among the plethora of medicinal plants available, *Rumex dentatus* L. emerges as a traditional pharmacophore widely used for its potent medicinal properties against various diseases, including pneumonia, asthma, jaundice, and cancer [11]. *R. dentatus* L. is regarded for its antimicrobial, anticancer, anti-inflammatory, and antioxidant activities. Being rich in bioactive compounds like anthraquinones and flavonoids, this plant has garnered attention for its therapeutic potential [12].

Rumex dentatus L. contains diverse secondary metabolites exhibiting anticancer properties. These compounds exert their effects through multiple mechanisms, including apoptosis induction, cell cycle arrest, and modulation of oxidative stress, thus presenting potential as natural anticancer agents. Anthraquinones (e.g., emodin, chrysophanol) induce apoptosis and inhibit cancer cell proliferation. Flavonoids (e.g., kaempferol, quercetin) reduce cancer cell survival, promote apoptosis, and impede metastasis. Tannins (e.g., ellagitannins) scavenge free radicals and modulate pathways involved in cancer cell growth. Saponins (e.g., dioscin) trigger apoptosis and enhance the immune response against tumors. Phenolic acids (e.g., caffeic acid) inhibit tumor growth by preventing angiogenesis and mitigating oxidative stress [13].

Furthermore, *R. dentatus* L. is recognized for its abundance in secondary metabolites with anticancer effects, acting through multiple molecular signaling pathways such as apoptosis, p53-dependent autophagy, cell cycle regulation, PI3K-AKT, JAK-STAT, MAPK-ERK, MAPK-JNK/p38, Wnt, and immunomodulation (TNF-α-NF-κB) pathways [14].

The advent of huge biomedical data has facilitated the emergence of network pharmacology as a systematic approach for analyzing drug targets. This has led to a shift in the drug discovery approach from the traditional "one target, one drug" model to a more complex "network target, multi-component therapy" approach [15]. As compound molecules bind directly to one or more cell proteins, these target proteins subsequently influence other related proteins [16].

Aligned with this transformation, in this study, we explored the anticancer cellular responses to cisplatin alone or in combination with *R. dentatus* L. in vitro, focusing on their effects on apoptosis and autophagy in the tongue carcinoma cell line (HNO97) and elucidating the underlying molecular mechanisms. Network pharmacology analysis was employed to establish how *R. dentatus* L. phenolic aglycones can potentially target tongue carcinoma. Assessment of the drug-likeness of bioactive compounds was carried out using computed molecular parameters related to their absorption, distribution, metabolism, and excretion (ADME).

## Materials and methods

We followed all the guidelines, approved by the Research Ethical Committee at the Faculty of Pharmacy, AlSalam University in Egypt (SUEP/REC/03/24/02/001).

### Plant collection, extraction, and phytochemical analysis

#### Plant collection and extraction

The aerial parts of *R. dentatus* L. in the growth stage 3, were collected from Kafr El-Sheikh city in May 2021. The plant was identified by Professor Yaseen Alsodany, Botany Department, Faculty of Science, Kafr ElSheikh University, Egypt, and a voucher specimen (ID 21–5-A2) was deposited at the herbarium of Pharmacognosy Department, Faculty of Pharmacy, Tanta University. The plant was dried in the shade for a week and the remaining moisture was eliminated by oven-drying at 40 °C for 4 h. The dried plant was ground into fine powder to obtain 500 g which was macerated in ethyl acetate (2 L, analytical grade) for 24 h, then filtered the process was repeated 3 times and the filtrates were combined. The combined filtered extract was evaporated under vacuum using a rotary evaporator at 55 °C to get a dark reddish viscous residue (40.20 gm, 4% yield). The residue was dissolved in methanol (0.5 L, analytical grade) and extracted with petroleum ether (analytical grade) till exhaustion (5. L, 3 times). The remaining methanol extract was evaporated under a vacuum using a rotary evaporator at 50 °C to get a dark reddish powder residue (20.03 gm, 2% yield). The last residue was used for the phytochemical analysis and biological investigation.

#### Phytochemical analysis using UPLC-ESI–MS/MS analysis

The extracted residue was characterized chemically to identify the phytochemicals included. The UPLC-ESI/MS/MS analysis, in both positive and negative ion modes, followed a published protocol [17] employing the same conditions, parameters, solvent system, and apparatus.

### Drugs

Cisplatin (Unistin®) at a concentration of 1 mg/mL was obtained from EMIC United Pharmaceuticals, Egypt.

### Cell Line

The human tongue carcinoma cell line (HNO-97) and Human Oral Epithelial Cell Line (OEC) were procured from Nawah-Scientific Research Centre (Almokattam Mall, Cairo, Egypt). Cells were cultured in Dulbecco’s modified Eagle’s medium (DMEM) (Lonza Group Ltd., Switzerland), supplemented with 100 mg/mL streptomycin, 100 units/mL penicillin, and 10% heat-inactivated fetal bovine serum (Sigma-Aldrich, USA) in a humidified, 5% CO_2_ atmosphere at 37 °C.

### Biological assays and anticancer activity evaluation

#### Cytotoxicity assay

A cytotoxicity assay was conducted using the Sulforhodamine B (SRB) method to evaluate the impact of the extract of *R. dentatus* L. on the proliferation of HNO-97 and Human Oral Epithelial Cell Line (OEC), a normal oral epithelial cell line. Cells (5 × 10^3 cells) were seeded in 96-well plates and incubated in complete media for 24 h. Subsequently, the cells were treated with either vehicle, cisplatin, or the plant extract at varying concentrations (0.01, 0.1, 1, 10, 100 µg/mL) and incubated for 72 h.

Following the 72-h exposure period, the cells were fixed by replacing the media with 10% cold trichloroacetic acid (TCA) and incubated at 4°C for 1 h. After removing the TCA solution, the cells were washed multiple times with distilled water. Next, they were stained with SRB solution (0.4% w/v) in the dark at room temperature for 10 min. Following staining, the plates were washed with 1% acetic acid and allowed to air-dry overnight. To solubilize the protein-bound dye, Tris base solution (10 mM) was added, and the absorbance of the plates was measured at 540 nm using a microplate reader. The half-maximal inhibitory concentration (IC_50_) of the treated groups was determined by comparison with the negative control group (media without drugs) [18].

#### Cell cycle analysis

Following treatment with either the plant extract or cisplatin alone or in combination, at a fixed drug concentration ratio of 1:1 for 48 h, cultured cells (10^5^ cells) were harvested by trypsinization and washed twice with ice-cold PBS (pH 7.4). The collected cells were then suspended in 2 mL of 60% ice-cold ethanol and incubated at 4 °C for 1 h to facilitate fixation. After fixation, the cells were washed twice with PBS (pH 7.4) and re-suspended in 1 mL of PBS containing 50 µg/mL RNAase A and 10 µg/mL propidium iodide (PI). Following a 20-min incubation in the dark at 37 °C, the cells were subjected to DNA content analysis using flow cytometry with an FL2 (λex/em 535/617 nm) signal detector (ACEA Novocyte™ flow cytometer, ACEA Biosciences Inc., USA). Each sample acquired 12,000 events, and cell cycle distribution was determined using ACEA NovoExpress™ software (ACEA Biosciences Inc., USA) [13].

#### Analysis of apoptosis

To differentiate between necrotic and apoptotic cell populations, an Annexin V-FITC apoptosis detection kit (Abcam Inc., Cambridge Science Park, Cambridge, UK) was employed, utilizing two fluorescent channels in flow cytometry analysis. Following treatment with the test compounds for 48 h, cultured cells (10^5^ cells) were harvested by trypsinization and washed twice with ice-cold PBS (pH 7.4). Subsequently, the cells were incubated in the dark with 0.5 mL of Annexin V-FITC/PI solution for 30 min at room temperature, according to the manufacturer's protocol.

After staining, the cells were analysed using an ACEA Novocyte™ flow cytometer (ACEA Biosciences Inc., San Diego, CA, USA), with FITC and PI fluorescent signals detected using FL1 and FL2 signal detectors, respectively (λex/em 488/530 nm for FITC and λex/em 535/617 nm for PI). Compensation was appropriately performed for the Annexin V/PI assay. Specifically, single-stain controls for both Annexin V (FITC) and PI (PE) were utilized to adjust for spectral overlap between these fluorophores. This compensation was conducted using [ACEA NovoExpress™ software (ACEA Biosciences Inc., San Diego, CA, USA), ensuring that the FITC signal did not bleed into the PE channel, and vice versa.

The dot plots presented in Figure B demonstrate distinct populations for live, apoptotic, and necrotic cells, with well-separated quadrants, confirming the adequacy of the compensation. Consequently, it is posited that the current data accurately reflect the apoptosis profiles of the cells in each treatment group. Single-stained controls were used to perform compensation and to generate a compensation matrix correcting for spectral overlap between detection channels. Experimental sample acquisition of 10,000 events per sample was performed. Gated cell populations based on the healthy cells:

- Viable cells (Healthy): Annexin V-FITC negative, PI negative
- Early apoptotic cells: Annexin V-FITC positive, PI negative
- Late apoptotic cells: Annexin V-FITC positive, PI positive
- Necrotic cells: Annexin V-FITC negative, PI positive

An isolated background signal of APC fluorescence using the histogram-gated population from the unstained tube was performed. Positive FITC and/or PI cells were quantified using quadrant analysis and calculated using ACEA NovoExpress™ software (ACEA Biosciences Inc., San Diego, CA, USA) [19]. Acquisition of 12,000 events per sample was performed, and positive FITC and/or PI cells were quantified using quadrant analysis and calculated using ACEA NovoExpress™ software (ACEA Biosciences Inc., San Diego, CA, USA) [19].

#### Autophagy assay

During autophagy, autophagosomes merge with lysosomes to create auto phagolysosomes, which can be visualized and stained with acridine orange. To quantitatively evaluate autophagic cell death in the HNO-97 cell line, acridine orange lysosomal stain coupled with flow cytometric analysis was utilized following treatment with test compounds at their IC_50_ for 48 h.

Cells (10^5^ cells) were harvested by trypsinization and washed twice with ice-cold PBS (pH 7.4). Subsequently, the cells were stained with acridine orange (10 µM) and incubated in the dark at 37 °C for 30 min. After staining, the cells were analysed using an ACEA Novocyte™ flow cytometer (ACEA Biosciences Inc., San Diego, CA, USA), with acridine orange, fluorescent signals detected using the FL1 signal detector (λex/em 488/530 nm). Acquisition of 12,000 events per sample was performed, and net fluorescent intensities (NFI) were quantified using ACEA NovoExpress™ software (ACEA Biosciences Inc., San Diego, CA, USA) [20].

#### Quantitative Reverse Transcription-Polymerase Chain Reaction (RT-qPCR)

Quantitative real-time polymerase chain reaction (RT-qPCR) was conducted to assess the impact of cisplatin, the extract of *R. dentatus* L., or their combined treatment on the relative gene expression levels of Bcl2, p53, and ATG7. Upon reaching 80% confluency in tissue culture flasks, cells were exposed to the IC_50_ concentration of *Rumex* or cisplatin, or a combination of both at a 1:1 ratio. Following 48 h of incubation, cells were collected by scraping.

Total RNA was extracted from the collected cells using the RNeasy Mini kit (Qiagen®, USA), by the manufacturer's instructions. Subsequently, the extracted RNA was reverse transcribed into complementary DNA (cDNA) using the Quantitect reverse transcription kit (Cat # 205,311) (Qiagen®, Germany). The resulting cDNA was then amplified using the SensiFAST™ SYBR No-ROX kit (Bioline, USA), following the manufacturer's protocol.

BLAST (available at: www.ncbi.nlm.nih.gov/blast/Blast.cgi ) was utilized to compare the primer and template sequences to other known sequences to check that they were unique.

The primer sequences utilized for amplification are:

GAPDH: (F: ATGGGGAAGGTGAAGGTCG, R: GGGGTCATTGATGGCAACAATA),

P53: (F: GAGAATCTCCGCAAGAAAGG, R: CTCATTCAGCTCTCGGAACA),

Bcl2: (F: CTTGACAGAGGATCATGCTGTAC, R: GGATGCTTTATTTCATGAGGC),

ATG7: (F: AGGAGATTCAACCAGAGACC, R: GCACAAGCCCAAGAGAGG).

The cycle threshold (CT) of each sample was compared to that of the positive control group using the formula (2^−ΔΔct^), with the relative expressions expressed as fold change. GAPDH was employed as the housekeeping gene, as per the method described by Livak and Schmittgen (2001) [21].

### Statistical analysis

The experiments were conducted in triplicates, and the results are presented as mean ± standard deviation (SD). Statistical analysis was performed using GraphPad Prism 6 software for data analysis and Microsoft Excel for graph plotting. Student's t-test was employed for comparing two samples, while one-way analysis of variance (ANOVA) followed by Tukey's post hoc test was utilized for comparisons involving more than two samples. A *p*-value of less than 0.05 was considered statistically significant.

### Computational study

#### Network pharmacology-based analysis

Analysis and identification of *R. dentatus* L. extract by using UPLC-PDA-MS/MS resulted in the identification of 14 bioactive compounds. In the current study we selected three of those bioactive compounds to screen their potential effect on tongue carcinoma. The components was determined using an in silico integrative model that focused on Absorption, Distribution, Metabolism, and Excretion (ADME), utilizing the Traditional Chinese Medicine Systems Pharmacology Database (TCMSP, http://tcmspw.com/tcmsp.php, version 2.3).

The screening process of involved bioactive compounds was based on the determination of their oral bioavailability (OB) and drug-likeness (DL) criteria. High OB values often play a critical role in drug development. Components with OB ≥ 30% were selected as potential active compounds for further analysis. The concept of DL is ambiguous because it indicates the similarity between the components and known drugs. Compounds exhibiting DL properties have the potential to become drugs but are not drugs themselves. A DL value greater than 0.18 indicates that the compound is chemically suitable for drug development purposes. Selected compounds are two flavonoids, quercetin and taxifolin and the anthraquinone emodin. The steps of the computational study were as follows:

(1) Identification of the human genes targeted by the selected bioactive compounds through the utilization of open databases (2) Establishing connections between the potential genes and the target disease (3) Analyses of protein–protein interactions (PPIs); and (4) Identification of the pathways associated with the target disease using public databases.

PubChem (https://pubchem.ncbi.nlm.nih.gov/) data base was used to collect chemical information about these compounds including their Canonical SMILES, and CID number.

#### Bioactive compounds-target network construction

Binding database (https://www.bindingdb.org) was utilized to search for the target genes associated with the selected bioactive compounds. This database is a platform for investigating established interactions between small molecules and proteins. This study compiled a final list of genes associated with selected bioactive compounds, considering a confidence score of ≥ 0.7 based on the binding database. This threshold indicates high-confidence interactions between bioactive compounds and proteins. Gene information, encompassing gene ID and name, was validated in the Uniport database (www.uniprot.org/help/uniprot ), with a species restriction set to "Homo sapiens." obtained results were utilized for the construction of compound-target (C-T) network using Cytoscape 3.10.1.

#### Target screening of cisplatin and tongue cancer

Fundamental details about cisplatin were retrieved from the PubChem database. The targets of cisplatin were compiled from various sources, including SuperPred and GeneCards, (https://www.genecards.org/) with a subsequent screening for duplicate results. Human gene names were cross-referenced in the Uniport database.

The term "tongue cancer" was entered into The Human Gene Database (GeneCards) to retrieve targets associated with it. The human gene names were then cross-referenced using the Uniport database. Subsequently, the identified targets of selected bioactive compounds, cisplatin, and tongue cancer were intersected, and a Venn diagram was generated to extract the shared target genes.

#### Protein–Protein Interaction (PPI) Network

A PPI network was constructed for shared target genes. These targets were input into the STRING database, with the species specified as Homo sapiens and medium confidence set to 0.4; other settings were maintained at their default values. The resulting data were, subsequently imported into Cytoscape software to construct the PPI network.

### Signalling pathway analysis

Gene Ontology (GO) and Kyoto Encyclopaedia of Genes and Genomes (KEGG) pathway enrichment analyses for shared target genes were carried out using the Shiny Go database (http://bioinformatics.sdstate.edu/go76). This aimed to identify relevant pathways and GO terms, encompassing biological process (BP), molecular function (MF), and cellular component (CC) categories. Pathways and GO terms reaching significance with a threshold of *p* < 0.05 were considered and retained. Additionally, Bioinformatics (http://www.bioinformatics.com.cn/) was employed to visualize the results of GO and KEGG enrichment analyses in a bar graph and a bubble plot depicting signaling pathways.

### Investigation of drug-likeness and ADME properties

To assess drug-likeness and pharmacokinetic properties, the Swiss ADME database (http://www.swissadme.ch/index.php) was employed. Evaluation of the ligands' drug-likeness score was carried out following Lipinski's rule of five [22].

## Results

### Phytochemical analysis of the extract of *R. dentatus* L.

**Fig. 1:**
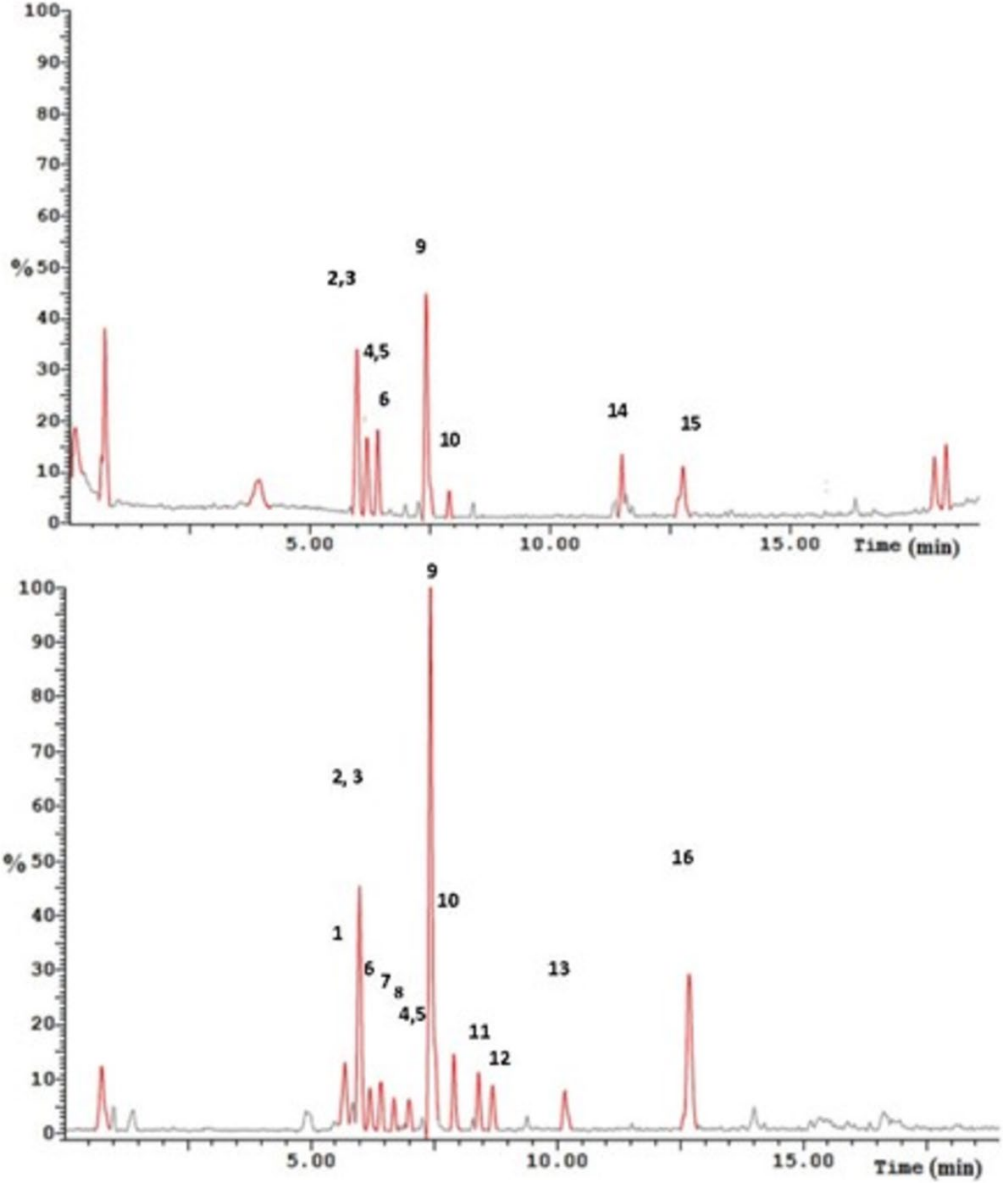
Base peak ion chromatograms of the UPLC-ESI–MS analysis of the extract of the aerial parts of *R. dentatus L*. in positive ion mode (top) and negative ion mode (bottom)

The UPLC-ESI–MS/MS analysis of the plant extract (Table 1, Fig. 1) resulted in the detection of 16 base peak ions of which 14 were identified based on their m/z ions and MS/MS fragmentation compared to the published datasets and databases. The identified compounds are phenolic and belong to the flavonoid and anthraquinone classes. The flavonoid compounds are flavonol and flavone skeletons.

**Table 1:** UPLC-ESI–MS/MS analysis of the extract of the aerial part of *R*. *dentatus* L

No	t_R_	m/z ESI +	m/z ESI-	MS/MS (ESI-)	Identification
1	5.69		303	303, 285, 275, 241, 217, 175, 151, 125	Taxifolin
2	5.98		303	303, 285, 275, 241, 217, 175, 151, 125	Taxifolin isomer
3	5.98		301	301, 273, 179, 151, 121, 107	Quercetin
4	6.19		301	301, 273, 179, 151, 121, 107	Quercetin isomer
5	6.19		299	284, 151	Kaempferol methyl ether
6	6.41	331	329	315, 314, 299, 271. 243, 227	Tricin
7	6.68		285	257, 243, 241, 213, 163, 151,	Kaempferol
8	7.00		299	284, 151	Kaempferol methyl ether isomer
9	7.42	289	287	151, 135, 125, 107	Eriodictyol
10	7.91	317	315	301, 272, 256, 151, 107	Isorhamnetin
11	8.40		301	301, 273, 179, 151, 121, 107	Quercetin isomer
12	8.68		285	285, 267, 241, 217, 199, 175, 151, 133	Luteolin
13	10.16		349	349	Unknown
14	11.50	247		247 (ESI +)	Unknown
15	12.67	271	269	256, 228, 225, 211, 143	Emodin
16	12.77		269	269, 240	Alo-emodin

Taxifolin, a flavanol with pseudomolecular ion [M-H]^−^ at m/z 303, was detected. The MS/MS profile showed the ions at m/z 285, 275, 241, 217, 175, 151, and 125 which resembles the published data for taxifolin [23].

Quercetin, a flavanol with pseudomolecular ion [M-H]^−^ at m/z 301, was detected. It was previously isolated from the roots of *R. dentatus* L. [24]. The MS/MS fragments at m/z 301, 273, 179, 151, 121, and 107 are by the published literature [23].

Tricin was identified in the extract. It was previously identified in *Rumex algeriensis*. It is a dimethoxyflavone with pseudomolecular ions [M + H]^+^ at m/z 331 and [M-H]^−^ at m/z 329. In the negative ion mode, the MS/MS fragments at m/z 315, 299, 271, and 243 were assigned to [M-H-CH_3_]^−^, [M-H-2CH_3_]^−^, [M-H-CH_3_-CO]^−^ and [M-H-2CH_3_−2CO]^−^ ions, respectively which is consistent with the literature [25].

Eriodictyol, a flavanone compound previously identified from *R. algeriensis*, was detected with pseudomolecular ions [M + H]^+^ at m/z 289 and [M-H]^−^ at m/z 287. In the negative ion mode, the MS/MS fragments at m/z 151, 135, 125, and 107 were assigned to [M-H-C_8_H_8_O_2_]^−^, [M-H-C_8_H_7_O_2_]^−^, [M-H-C_9_H_6_O_3_]^−^, [M-H-C_8_H_8_O_2_-CO_2_]^−^ which is following literature [25].

Isorhamnetin, a monomethoxy flavonol detected previously in *R. algeriensis*, was detected at m/z 317 and 315 for the pseudomolecular ions [M + H]^+^ and [M-H]^−^, respectively. The m/z 315, in the negative ion mode, fragmented to the ions at m/z 301 for [M-H-CH_3_]^−^, m/z 272 for [M-H-CH_3_-HCO]^−^, m/z 256 for [M-H-CH_3_-CO_2_]^−^, m/z 151 for [M-H-C_9_H_8_O_4_]^−^, and m/z 107 for [M-H-C_9_H_8_O_4_-CO_2_]^−^. This fragmentation pattern resembles that in the literature for isorhamnetin from *R. algeriensis* [25].

Kaempferol, a flavonol structure with a pseudomolecular ion [M-H]^−^ at m/z 285, was detected in the extract. The MS/MS fragmentation to yield the ions at m/z 257, 243, 241, 213, 163, 151, is identical to the published data [26].

Kaempferol methyl ether (kaempferide), a flavonol methyl ether with a pseudomolecular ion [M-H]^−^ at m/z 299, was identified in the extract. The MS/MS fragmentation ions at m/z 284 [M-CH_3_] and at m/z 151, are identical to the published literature [27].

Luteolin, a flavone compound with a pseudomolecular ion [M-H]^−^ at m/z 285, was detected. The MS/MS fragmentation ions at m/z 285, 267, 241, 217, 199, 175, 151, and 133, are identical to the published data [23].

Emodin and aloe-emodin revealed an [M-H]^−^ ion at m/z 269. For emodin, the characteristic [M-H–CO]^−^ ion at m/z 241 and [M-H-CO_2_]^−^ ion at m/z 225 were found in the MS–MS spectrum which is consistent with the literature [28]. However, for aloe-emodin the characteristic [M-H-CHO]^−^ ion at m/z 240 was detected in the MS/MS spectrum [28].

### In vitro cytotoxic activity

**Fig. 2:**
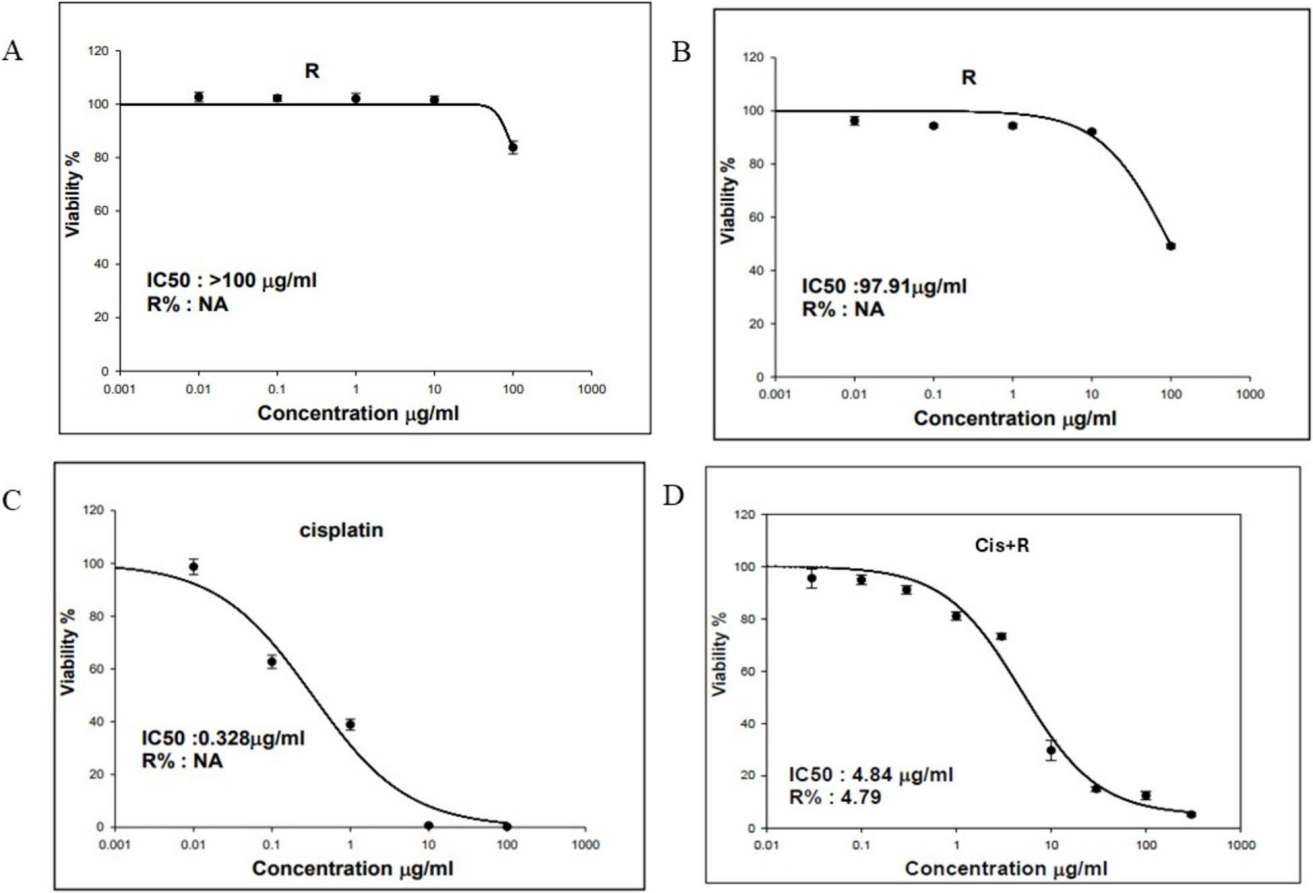
Cytotoxicity of *Rumex dentatus L*. (R) was assessed in vitro against **A**: Oral epithelial cell line (OEC); **B**: Tongue carcinoma cell line (HNO-97), **C**: Cytotoxicity of cisplatin against Tongue carcinoma cell line (HNO-97)

In vitro*,* cytotoxic activities of both *R. dentatus* L. extract and cisplatin against tongue carcinoma cell line (HNO-97) were evaluated by using SRB assay. Both *R. dentatus* L. extract and cisplatin inhibited the growth of the HNO-97 cells in a dose-dependent manner. However, notable, and strong effects were only exerted by *R. dentatus* L. at a concentration of 100 µg/mL (Fig. 2).

Atjanasuppat et al. [29] previously documented that plant extracts' antiproliferative effects are categorized based on their IC_50_ values into four classes. Extracts with IC_50_ ≤ 20 µg/mL are deemed active, those with IC_50_ between 20–100 µg/mL are considered moderately active, extracts with IC_50_ between 100–1000 µg/mL are labeled as weakly active, and finally, extracts with IC_50_ > 1000 µg/mL are characterized as inactive.

According to the results of the SRB assay, the calculated IC_50_ values of the prepared *R. dentatus* L. extract and cisplatin against the HNO-97 cell line were 97.9 and 0.328 μg/mL, respectively (Figs. 2B & 2C). Accordingly, we could say that *R. dentatus* L. extract showed moderate anti-proliferative activity against HNO-97 cells. Moreover, the cytotoxicity and safety of *R. dentatus* L. against normal oral epithelial cell line (OEC) was evaluated. Results showed that *R. dentatus* L. exhibited an IC_50_ > 100 μg/mL when tested on the OEC normal cell line as shown in Fig. 2A. This means that *R. dentatus* L. showed a weak cytotoxic activity against the OEC normal cell line indicating its safety.

To assess the synergy, additivity, or antagonism of the combination *Rumex* /Cisplatin the unified theory by Chou was employed for analysis in HNO-97 tongue carcinoma cells [30]. Combination index (CI) values were calculated using the following equation:

$$\mathbf{C}\mathbf{I}=(\mathbf{D}1)/(\mathbf{D}\mathbf{x})1+(\mathbf{D}2)/(\mathbf{D}\mathbf{x})2$$

The combination index (CI) theorem of Chou-Talalay provides a quantitative definition for additive effect (CI = 1), synergism (CI < 1), and antagonism (CI > 1) in drug combinations. The CI represents the natural law–based general expression of pharmacologic drug interactions. It has been demonstrated to be the most parsimonious method for quantifying synergism or antagonism. The simplicity of its equations, experimental designs, and data analysis contribute to efficiency, economy, and a reduction in the number of experimental animals or patients required for drug combination clinical trials. The combination index (CI) utilizes (D)1 and (D)2 representing the combination doses of drug 1 and drug 2 that achieve a 50% growth inhibition effect. Additionally, (Dx)1 and (Dx)2 denote the individual doses of drug 1 and drug 2, respectively, resulting in the same 50% growth inhibition effect. This index facilitates the classification of drug interactions as synergistic, additive, or antagonistic.

**Fig. 3:**
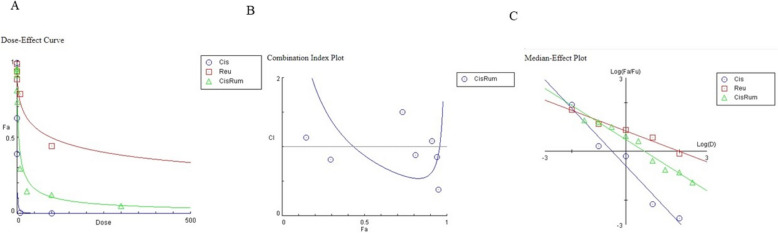
Dose Effect Curve and Combination Index Plot of combination treated group. **A** Dose–effect curves of Cis, R and their combination on HNO-97 cells, **B **Combination index plot of Cis + R in HNO-97 cells, **C** Median–effect plot of Cis, R and their combination on HNO-97 cells

According to the calculated combination index (CI) from the previous equation, a CI value of less than 1 confirms a synergistic interaction between the drugs in the combination. The impact of these combinations on cell proliferation was confirmed using Compusyn software (Version 1.0, Compusyn, Inc., Paramus, NJ, USA), which computed the CI values. Specifically, CI values below 1 indicate synergy, and in this study, the CI was calculated as 0.98, supporting the presence of a synergistic effect, as illustrated in Fig. 3.

### Cell cycle analysis

**Fig. 4:**
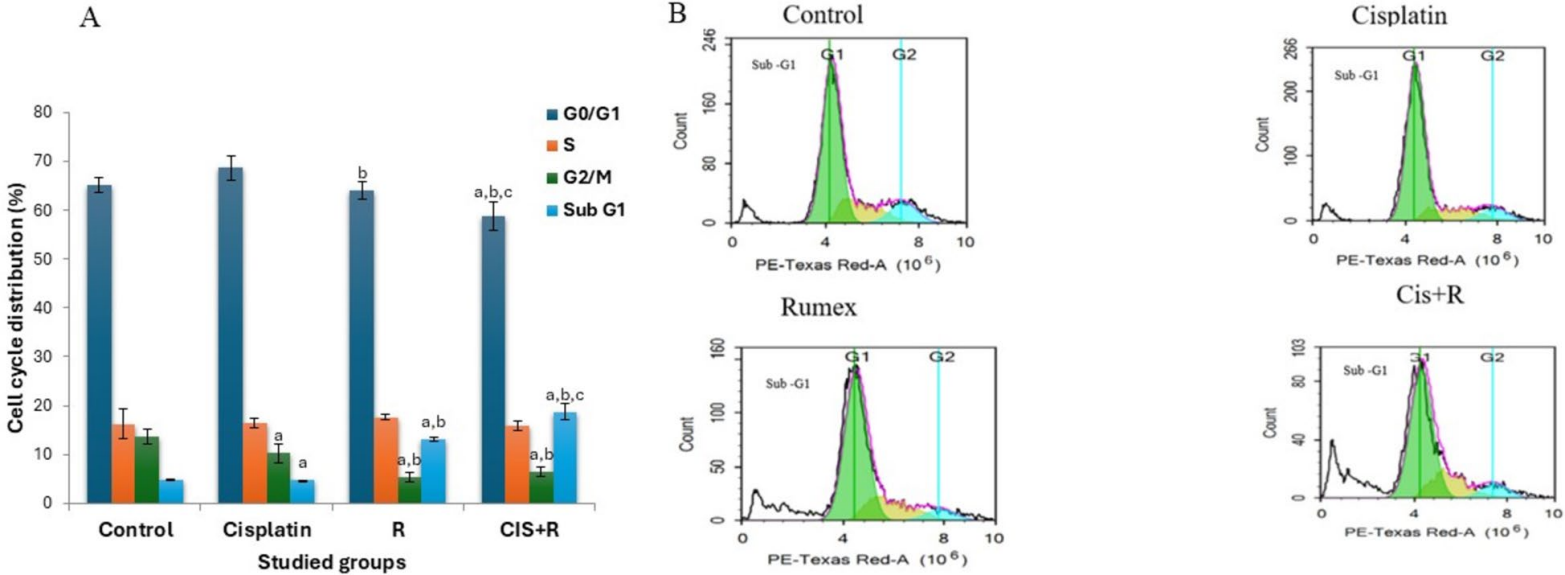
**A** Effects of *Rumex dentatus* (R) and cisplatin (CIS) or combination of both on sub-G1 phase, G0/G1 phase, S phase, and G2/M phase in HNO-97 cells after 48 h treatment. **B** Cell cycle analysis was performed using flow cytometry. The histograms show the correlation between DNA content, measured as PI fluorescence on the x-axis, and cell counts, represented on the y-axis, for various treatments. a: significant versus control, b: significant versus cisplatin, c: significant versus rumex. CIS: cisplatin, R: *Rumex dentatus L*., CIS + R: combination of cisplatin and *Rumex dentatus L*

Cell cycle distribution patterns were studied by using flow cytometric analysis against HNO-97 cells. Propidium iodide staining was employed for cell cycle analysis, and the percentage distribution of cells across various phases of the cell cycle was assessed following treatments with pre-determined IC_50_ values for *R. dentatus* L., cisplatin, and a combination of both for 48 h. Flow cytometry determined DNA content in the sub-G1 phase, G0/G1 phase, S phase, and G2/M phase at the end of the treatment. Figure 4A shows the effects of *R. dentatus* L. and cisplatin or a combination of both on the sub-G1 phase, G0/G1 phase, S phase, and G2/M phase in HNO-97 cells after 48 h treatment. Untreated cells showed a normal cell cycle with low sub-G1 DNA (Fig. 4). Results showed that treatment of HNO-97 cells with *R. dentatus* L. resulted in a significant increase in the sub-G1cell population (*P* < 0.05) accompanied by a decrease in the G0/G1 and G2/M phase cell population compared to the control group. Accumulation of cells in the subG1 phase indicates apoptotic cell death. Moreover, treatment of HNO-97 cells with a combination of *R. dentatus* L. and cisplatin showed a dramatic increase (*p* < 0.05) in the sub-G1 cell population compared to both control and either *R. dentatus* L. or cisplatin alone (Fig. 4B).

### Flow cytometric detection of apoptosis by annexin V-FITC/PI binding assay

**Fig. 5:**
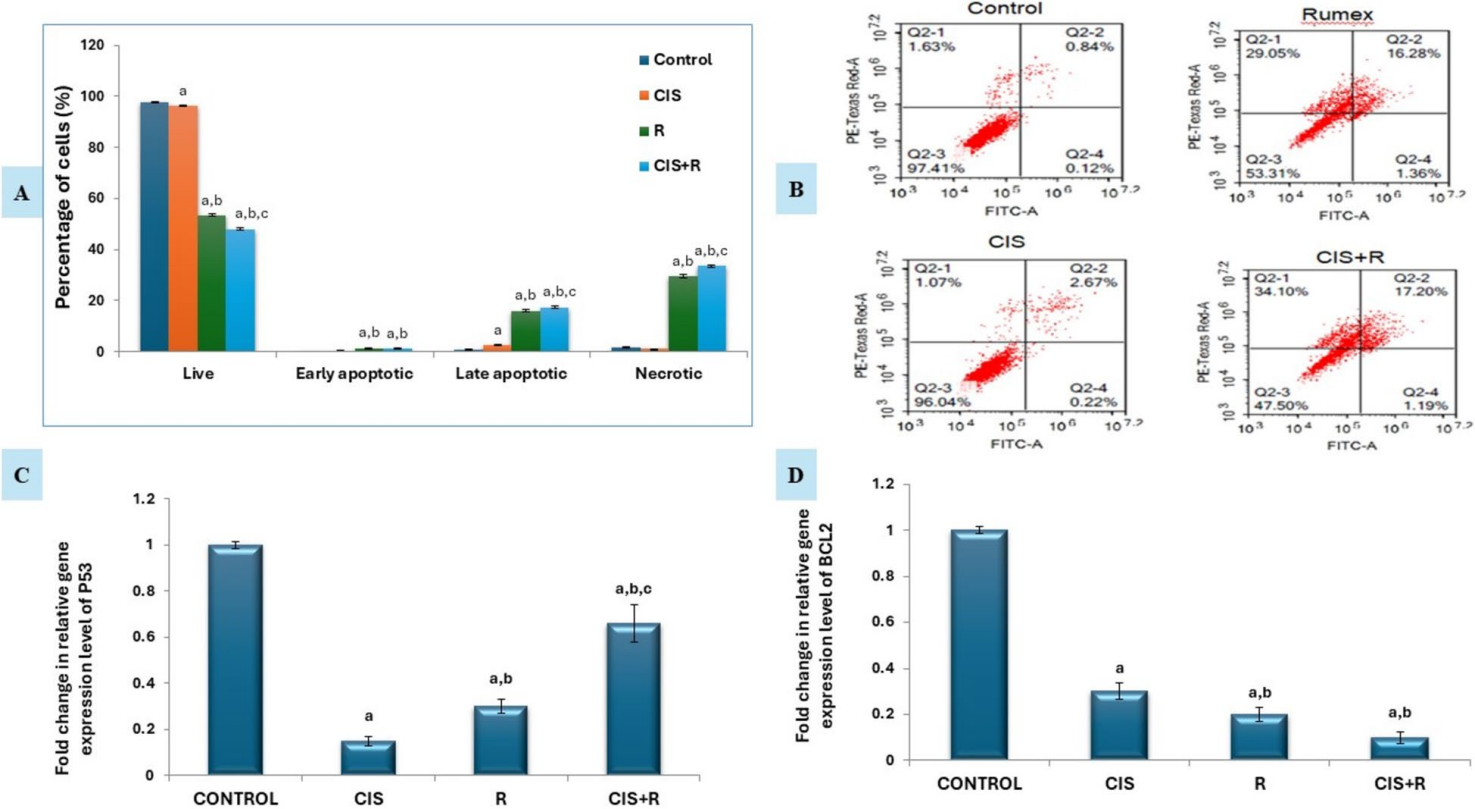
Effect of Rumex dentatus L. extract treatment either alone or in combination with cisplatin on apoptosis. Evaluation of apoptosis using Annexin V-PI double staining. **A** Quantification of the percentage of cells in different stages of apoptosis as determined by flow cytometry. **B** Representative flow cytometry dot plots of apoptosis. In all four plots, viable cells are observed in the left lower quadrant (FITC-/PI-), early apoptotic cells in the right lower quadrant (FITC + /PI-), and late apoptotic cells in the right upper quadrant (FITC + /PI +). **C** Fold change of P53 relative gene expression level by qPCR. **D** Fold change of BCL2 relative gene expression level by qPCR. a: significant versus control, b: significant versus cisplatin, c: significant versus Rumex. CIS: cisplatin, R: Rumex dentatus L., CIS + R: combination of cisplatin and Rumex dentatus L

We employed the Annexin V-FITC/PI double-labelling technique in our study to assess the number of apoptotic cells through flow cytometry analysis following treatment with pre-determined IC_50_ concentrations. The Annexin V-FITC/PI method can detect phosphatidylserine (PS), recognized as a biomarker for early apoptosis. As the apoptosis process advances, causing cell membrane breakdown, propidium iodide (PI) can then stain DNA. Flow cytometric data revealed that *R.* induced apoptosis in HNO-97 cells. Both the *R. dentatus* L. treated group and the co-treated group showed a significant increase (*p* < 0.05) in the percentage of early apoptotic cells compared to the control and cisplatin-treated group. Moreover, the combination of *Rumex* and cisplatin showed a significant increase (*p* < 0.05) in the percentage of late apoptotic cells compared to other groups indicating that *R. dentatus* L. augmented the cytotoxicity of cisplatin (Fig. 5A, B).

### Effect of *R. dentatus* L. extract treatment either alone or in combination with cisplatin on gene expression of Bcl2 protein and p53 Protein in HNO-97 cells

HNO-97 cells treated with a combination of *R. dentatus* L. extract and cisplatin showed a significant increase (*p* < 0.05) in gene expression of p53 protein in HNO-97 cells compared to cisplatin and rumex mono-treated groups. On the other hand, results showed that in HNO-97 cells treated with a combination of both cisplatin and *Rumex dentatus* L. extract, gene expression of BCL2 protein was significantly decreased (*p* < 0.05) compared to cisplatin alone (Figs. 5C&D).

### Effect of *R. dentatus* L. extract treatment either alone or in combination with cisplatin on gene expression of autophagy protein ATG7 in HNO-97 cells

**Fig. 6:**
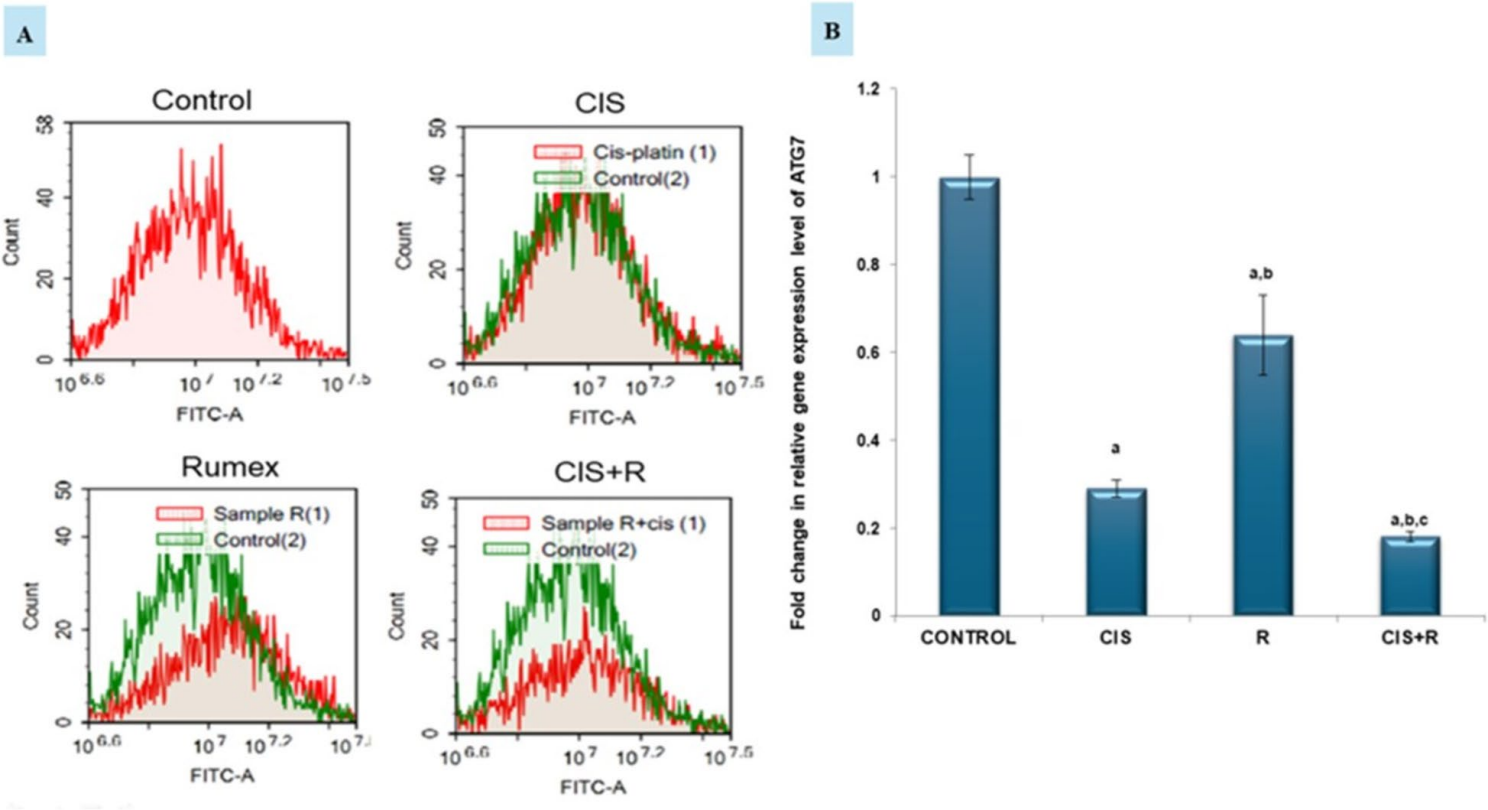
Autophagic cell death evaluation of the effect of Rumex dentatus L. extract treatment either alone or in combination with cisplatin in HNO-97 cells. **A** Representative histograms for quantifying autophagy using acridine orange lysosomal stain. **B** Fold change of autophagy-related gene relative gene expression level. a: significant compared to control, b: significant compared to cisplatin, c: significant compared to rumex. CIS: cisplatin, R: Rumex dentatus L., CIS + R: combination of cisplatin and Rumex dentatus L

Single treatment of HNO-97 cells with *R. dentatus* L. extract or cisplatin showed a significant decrease (*p* < 0.05) in gene expression of autophagy protein ATG7 in HNO-97 cells by (36% and 71%) respectively versus the control group. Moreover, treatment of HNO-97 cells with a combination of *R. dentatus* L. extract and cisplatin showed a significant decrease in gene expression of autophagy protein ATG7 in HNO-97 cells by (82%,37.9%,71.8%) compared to control (*p* < 0.05), cisplatin (*p* < 0.05) and *R. dentatus* L. treated groups. Our results were supported by autophagy assay (Fig. 6A&B).

### Network pharmacology

#### Bioactive chemical compounds in *Rumex dentatus* L.

To conduct our network computational study, we selected three bioactive chemical compounds contained in *R. dentatus* L. after we carried out phytochemical characterization of the extract. Selected compounds are two flavonoids, quercetin and taxifolin, and the anthraquinone emodin.

#### Bioactive compounds-target network

Identifying target genes associated with the bioactive compounds of *R. dentatus* L. was achieved using the binding database (https://www.bindingdb.org). A total of 154 target genes were selected. Taxifolin interacted with 55 targets, emodin interacted with 18 targets, and Quercetin interacted with 81 targets each possessing a confidence score of ≥ 0.7. The significance of a node within the interaction network increases with higher values in its degree, betweenness centrality, and closeness centrality. Using the merging option in Cystoscope software, 3.10.1., a merged network that combines each compound with identified target hits was formed. The target genes identified by the three bioactive compounds were combined and filtered to remove duplicates.

These genes were used to construct compound–gene networks, where the active identified compounds of each species were connected to corresponding annotated genes.

**Fig. 7:**
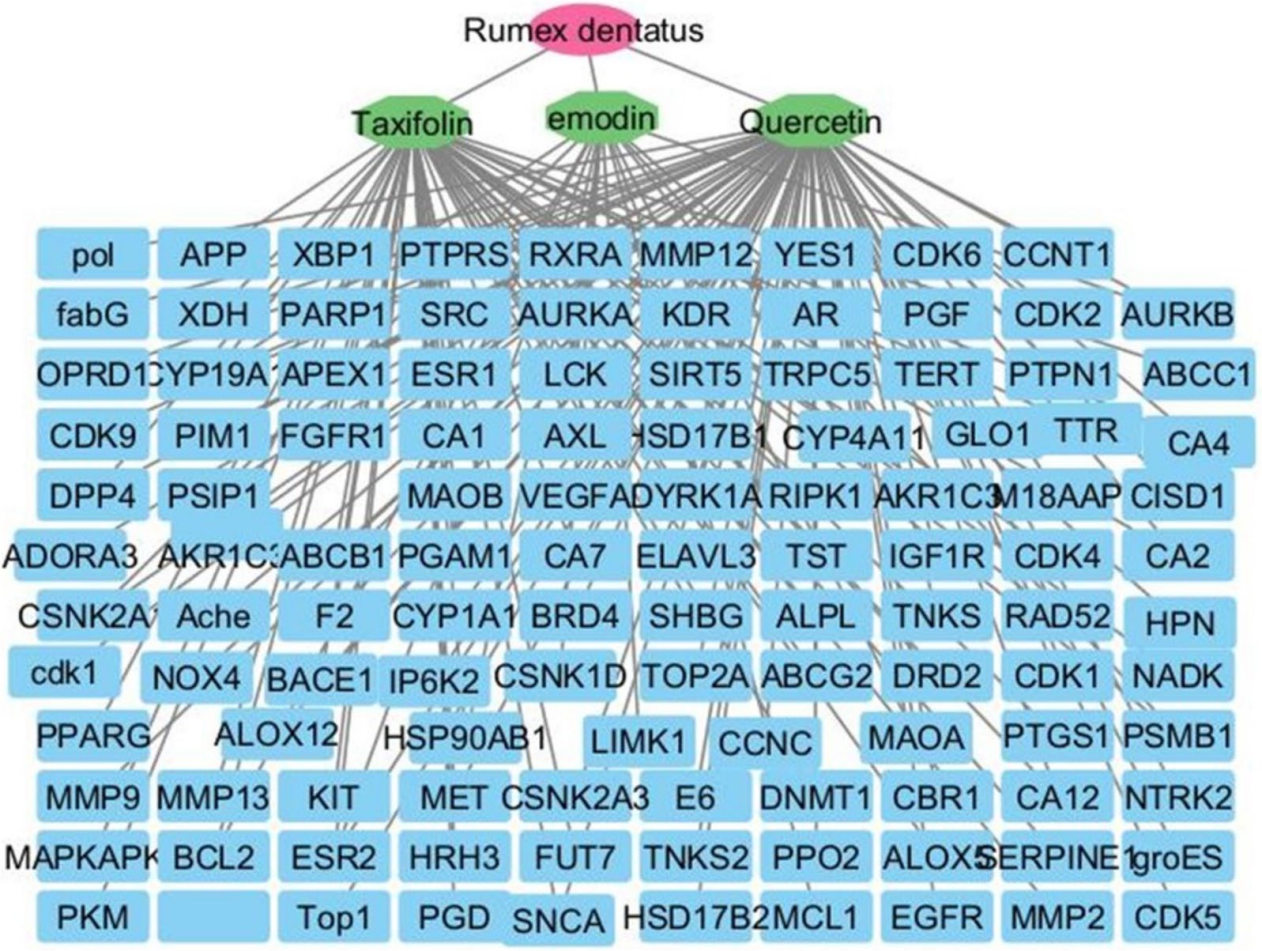
Network visualization depicts the three bioactive compounds identified from *Rumex dentatus* (Taxifolin, Emodin, and Quercetin) and their targets. The red circle denotes the plant species, the green octagons represent the bioactive compounds, and the blue rectangles represent the targets Edges represent interactions between bioactive compounds and targets. The merged network shows the relationship between compounds and targets

Specifically, for the compound’s quercetin, taxifolin, and emodin, their degrees were 55, 75, and 18, respectively. Additionally, their closeness centralities were 0.4876, 0.5841, and 0.3734, and betweenness centralities were 0.5069, 0.7222, and 0.13, respectively. In this network, taxifolin exhibits the highest degree, of closeness centrality, and betweenness centrality. The network consists of 3 bioactive compounds and involves 154 genes, totaling 120 nodes and 148 edges (Fig. 7).

#### Screening for targets of cisplatin and tongue cancer

**Fig. 8:**
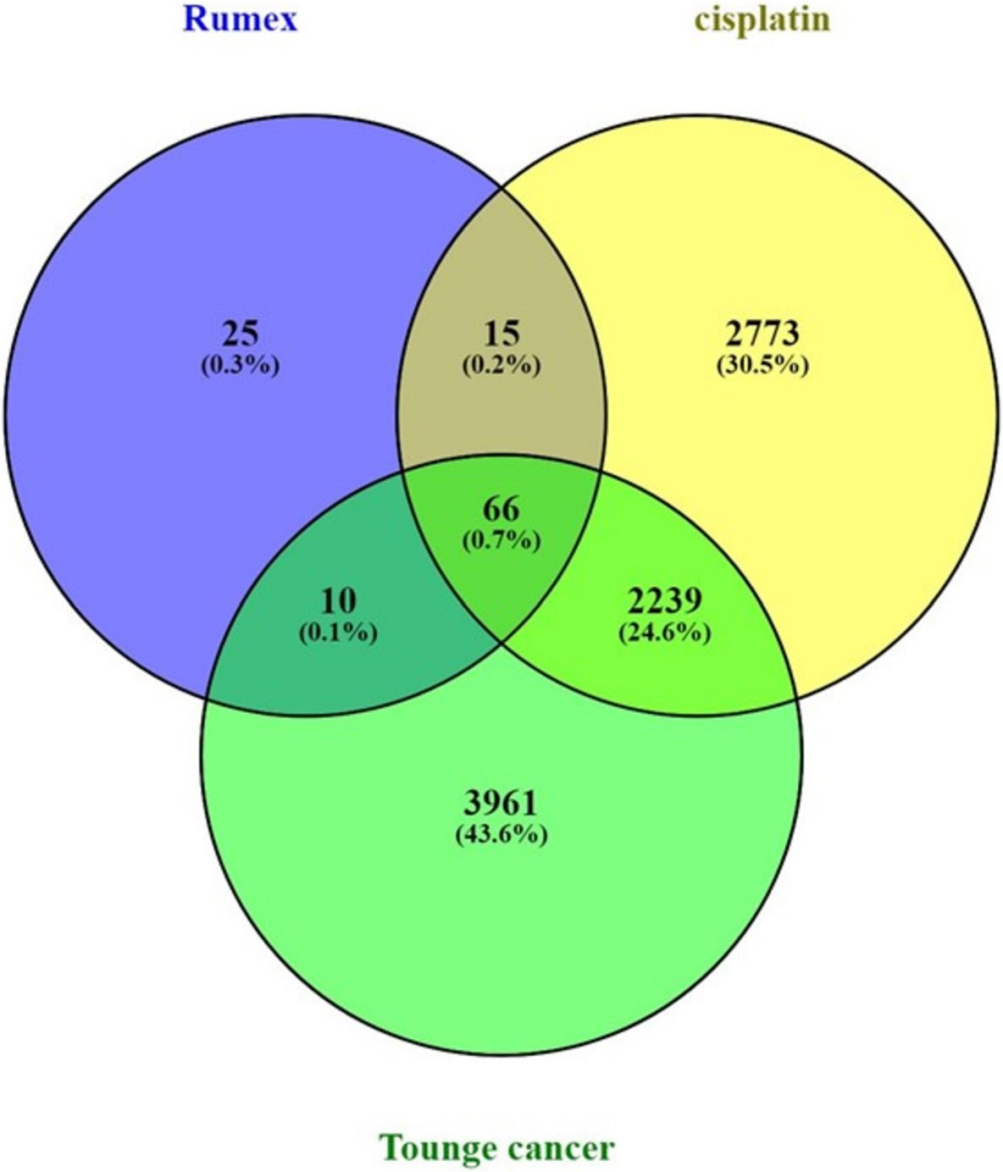
The Venn diagram depicts the overlapping target genes shared among the bioactive compounds of *R. dentatus*, tongue cancer, and cisplatin. The size of the circles corresponds to the number of target genes, with the green circle representing the target genes of tongue cancer, the blue circle indicating the target genes associated with the quantitatively determined selected bioactive compounds of R. dentatus, and the yellow circle representing the target genes of cisplatin. The overlapping region signifies the common target genes between the three

**Fig. 9:**
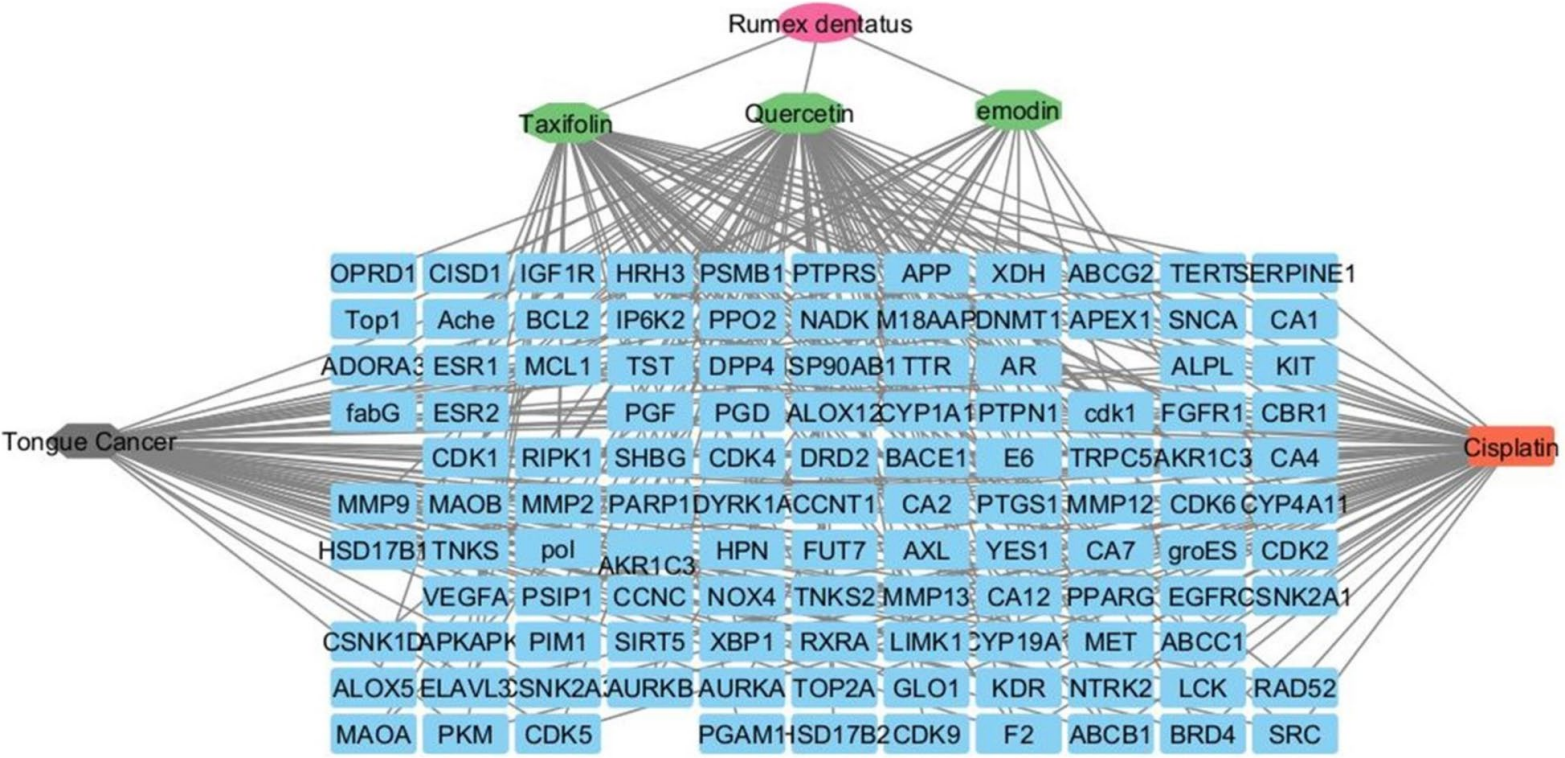
The Total Pharmacology Network depicts the three bioactive compounds of *Rumex dentatus*(taxifolin, quercetin, and emodin) as green octagons. Tongue cancer is represented by a gray hexagon, whereas cisplatin is represented by a red rectangle. The network also shows genes representing shared targets (blue rectangles)

**Fig. 10:**
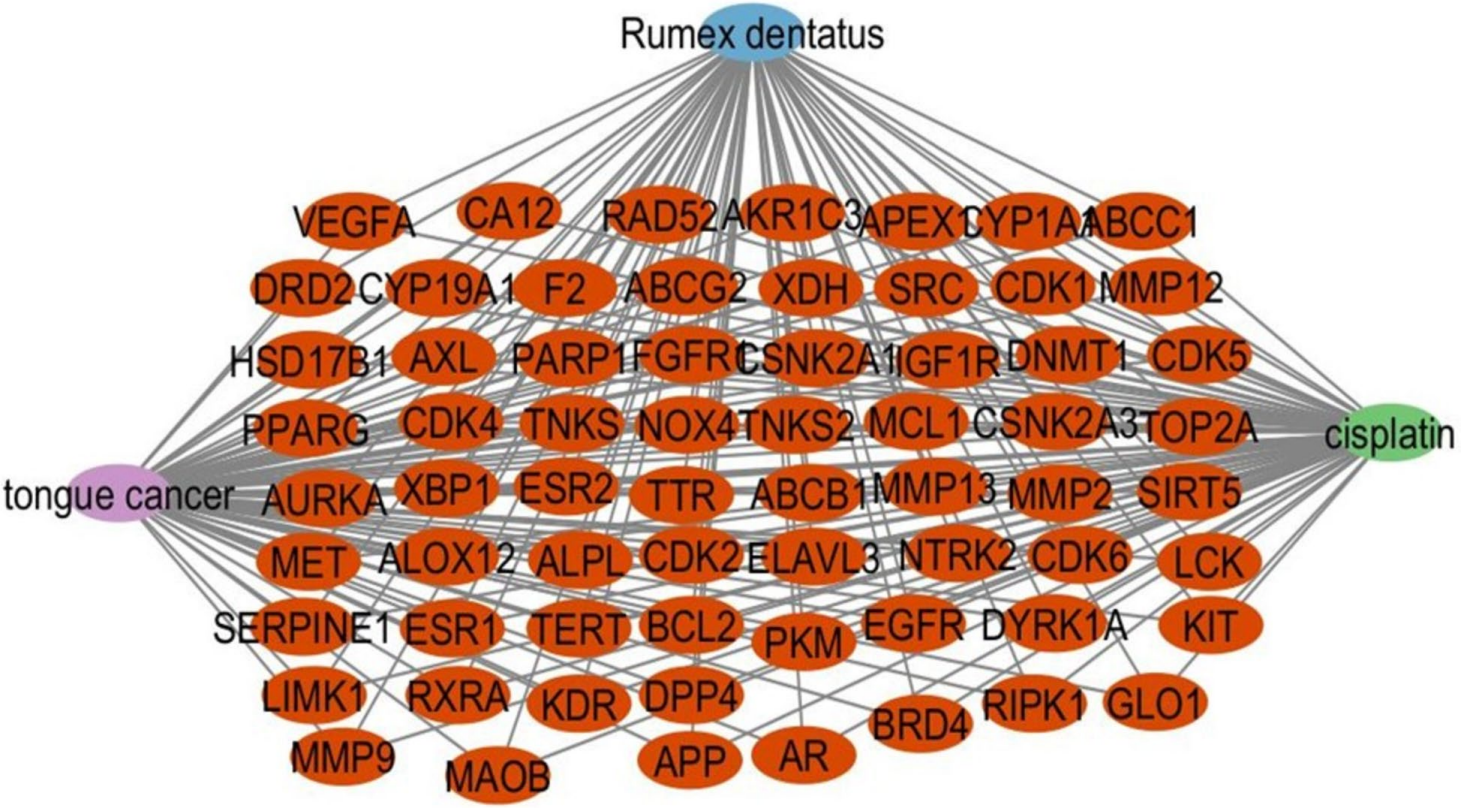
Total pharmacology network: The network integrates Rumex-selected bioactive compounds with genes associated with tongue cancer and the bioactive compounds of Rumex and cisplatin which identified the genes implicated in tongue cancer (in red oval shapes)

Cisplatin was associated with 5,039 targets. To identify tongue cancer-related targets, the keyword "tongue cancer" was utilized in the human gene database (Gene Cards), yielding 6276 targets. Subsequently, human gene names were updated using the Uniport database. The common target genes among selected bioactive compounds, cisplatin, and tongue cancer were then identified through the intersection of their obtained targets, and a Venn diagram was generated for this purpose. A total of 66 shared targets were identified (Fig. 8). Cytoscape 3.10.1 was used to construct a cisplatin-disease-common target network. Using the merging option in Cystoscope software, 3.10.1., a merged network has been created, combining the bioactive compounds-target network and the cisplatin-disease-common target network to induce total pharmacology network. This network consists of 122 nodes, with one node representing *Rumex dentatus*, three nodes representing the three selected bioactive compounds, one node representing cisplatin, one node representing tongue cancer, and 66 nodes representing shared targets. The remaining nodes represent non-shared targets of Rumex dentatus bioactive compounds (Fig. 9). We also used Cytoscape to construct a total pharmacology network this network integrates Rumex-selected bioactive compounds with genes associated with tongue cancer and the bioactive compounds of Rumex and cisplatin (66 genes determined by Venn diagram) which identified the genes implicated in tongue cancer (Fig. 10).

#### Protein–protein interaction network

**Fig. 11:**
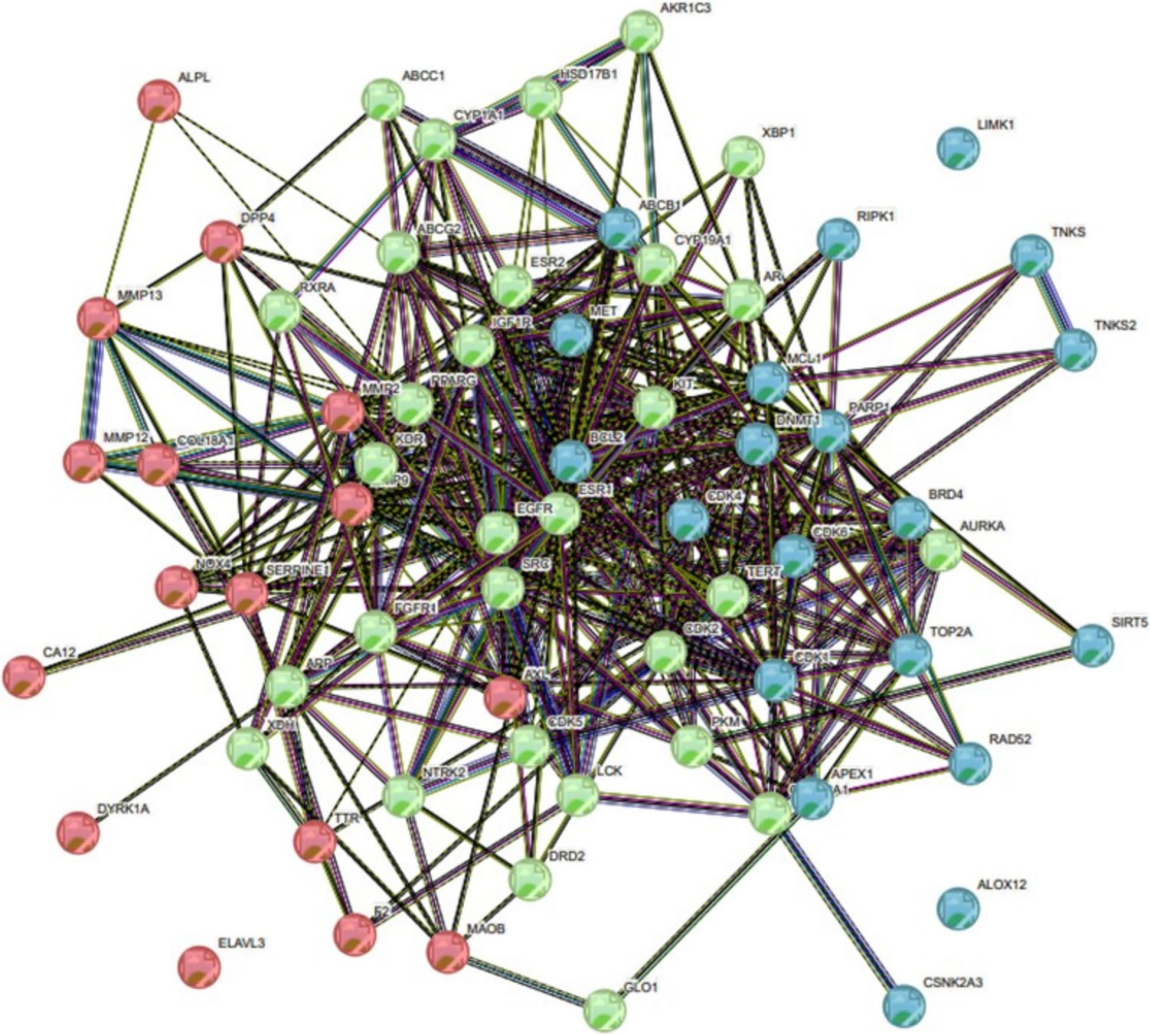
STRING Network Analysis Displaying Protein–Protein Interactions. Colored nodes represent query proteins and their first shell of interactions. A red node indicates Cluster 1, a green node represents Cluster 2, and a blue node signifies Cluster 3. Each node in the network corresponds to an individual protein, while the edges connecting them indicate specific protein–protein associations. The varying colors of the nodes reflect different levels of interaction. Filled nodes indicate that the structures are known or predicted. The edges illustrate functional protein associations, with the thickness of the lines representing the strength of these interactions

The STRING database played a crucial role in analyzing the interaction among 66 potential therapeutic targets. The resulting PPI network consisted of three clusters with 66 nodes and 451 edges, with an average node degree of 13.7. In this context, nodes symbolize individual proteins, and edges signify connections between them. The higher the degree value, the more pivotal the protein's role in the network. In cluster 1 average node degree is 10.4, in cluster 2 average node degree is 8.56, and in cluster 3 average node degree is 1.5 (Fig. 11). Table 2 shows a description of clusters in the PPI network including gene count protein names.

**Table 2:**
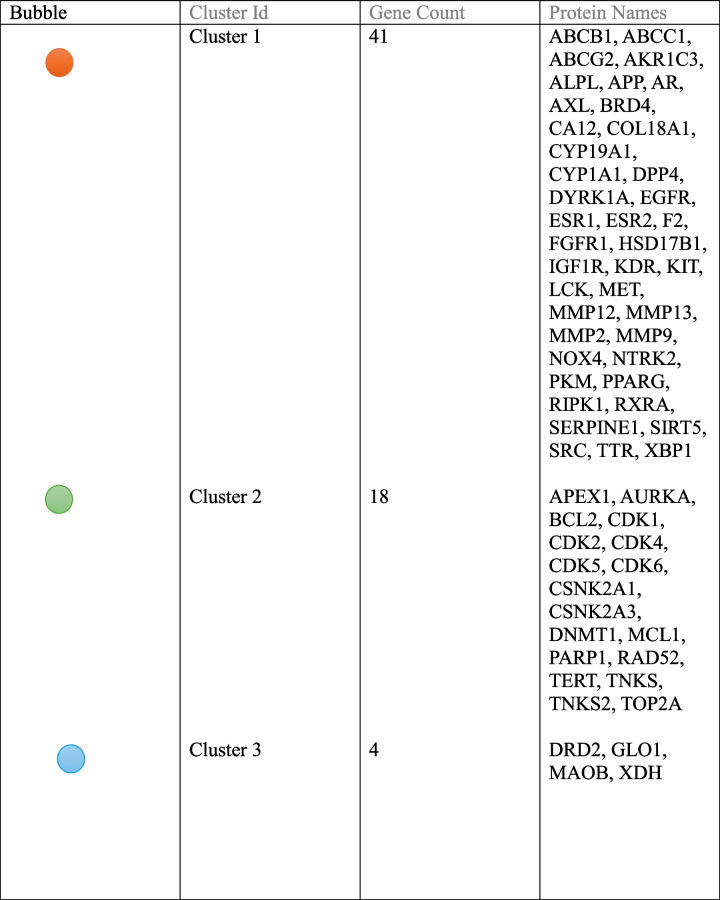
Description of clusters in PPI net work

**Fig. 12:**
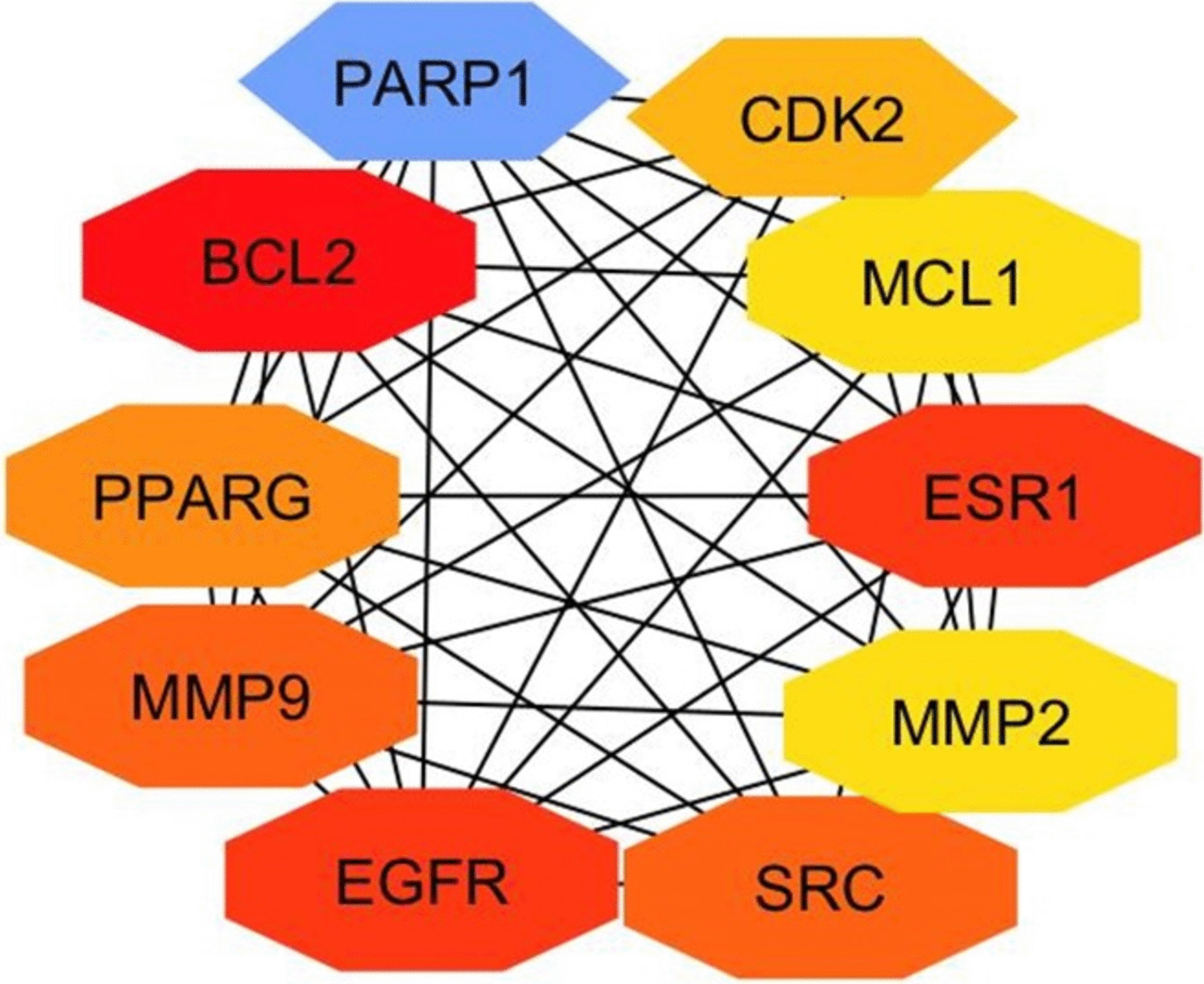
Hub target genes shared by Rumex, cisplatin, and tongue cancer. Hub genes were identified using the CytoHubba plugin in Cytoscape version 3.10.1, by loading a PPI network. The selected nodes are displayed in a color scheme that represents their level of importance, ranging from highly essential (red) to essential (yellow)

Through careful filtration 10 hub genes were identified. These genes exhibited exceptional network topology parameters, including degree, betweenness centrality (BC), and closeness centrality (Cc). According to these parameters ESR1, EGFR, and BCL2 are deemed core targets within the PPI network, highlighting their significance in potential therapeutic interventions which play an important role in the treatment of tongue cancer (Fig. 12) (Table 3).

**Table 3:** Degree of hub genes of PPI net work

Top 10 genes in PPI network ranked by Degree method	Name	Score
1	BCL2	42
2	EGFR	41
2	ESR1	41
4	MMP9	32
5	SRC	32
6	PARP1	32
7	PPARG	30
8	CDK2	26
9	MCL1	25
10	MMP2	25

### GO enrichment analysis

GO enrichment analysis was conducted on the 66 potential therapeutic targets of cisplatin and selected bioactive compounds expected to be therapeutic targets in tongue cancer. The resulting bar chart (Fig. 12a-b.) visually represents the top 10 Gene Ontology (GO) terms. In the chart, the length of the bars corresponds to the number of enriched target genes, while the color gradient from blue to red signifies a decrease in *p*-values, associated with that specific GO term. This implies a stronger association of the identified GO term with tongue cancer treatment compared to other GO terms.

**Fig. 13:**
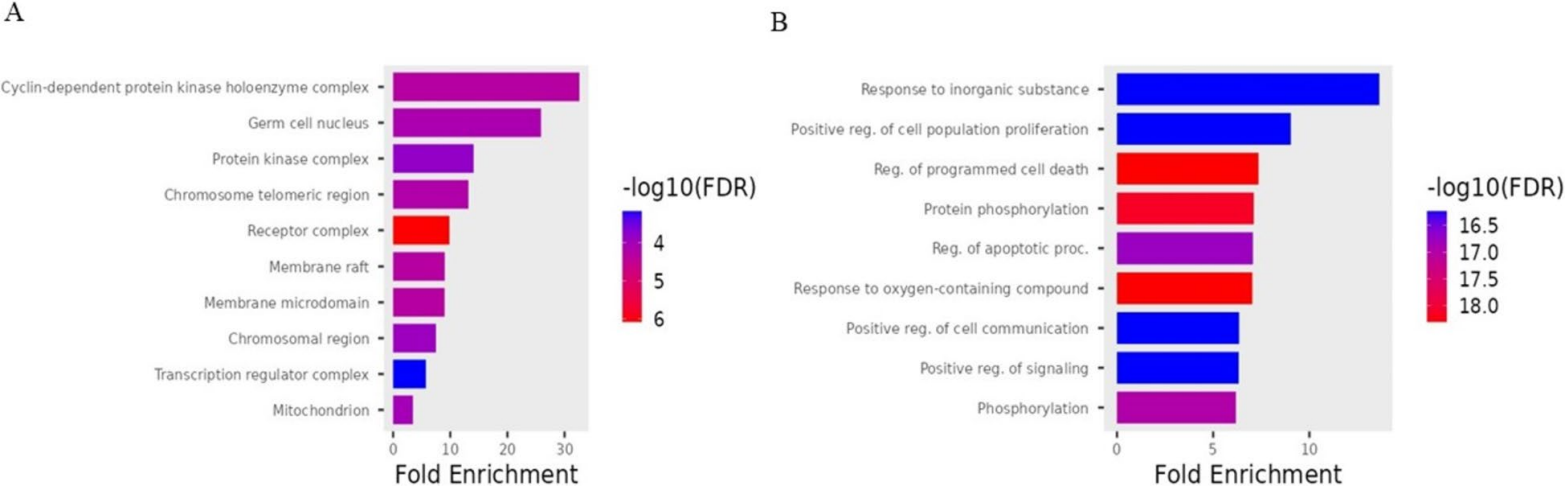
GO enrichment analysis **A** Represents cellular component **B** Represents biological function

Our analysis highlighted that the principal enriched Biological Process (BP) categories included are GO:1,901,700: Response to oxygen-containing compound, GO:0043067: Regulation of programmed cell death, GO:0010035: Response to inorganic substance, GO:0008284: Positive regulation of cell population proliferation, GO:0006468: Protein phosphorylation, GO:0010647: Positive regulation of cell communication, GO:0023056: Positive regulation of signaling, GO:0016310: Phosphorylation (Fig. 13). The analysis of cellular components (CC) unveiled that GO:0000307 cyclin-dependent protein kinase holoenzyme complex, GO:0043073 germ cell nucleus, GO:1,902,911 protein kinase complex, GO:0000781 chromosome telomeric region, GO:0043235 receptor complex, GO:0045121 membrane raft, GO:0098857 membrane microdomain, GO:0098687 chromosomal region, GO:0005667 transcription regulator complex GO:0005739 mitochondrion are considered the major enriched cellular component categories involved Fig. 13). Our analysis highlighted that the principal enriched molecular function (MF) categories included are GO:0097371: MDM2/MDM4 family protein binding, GO:0003684: damaged DNA binding, GO:0106310: protein serine kinase activity, GO:0004712: protein serine/threonine/tyrosine kinase activity, GO:0004674: protein serine/threonine kinase activity: GO:0004672: protein kinase activity, GO:0016773:phosphotransferase activity alcohol group as acceptor.

### KEGG pathway enrichment analysis

**Fig. 14:**
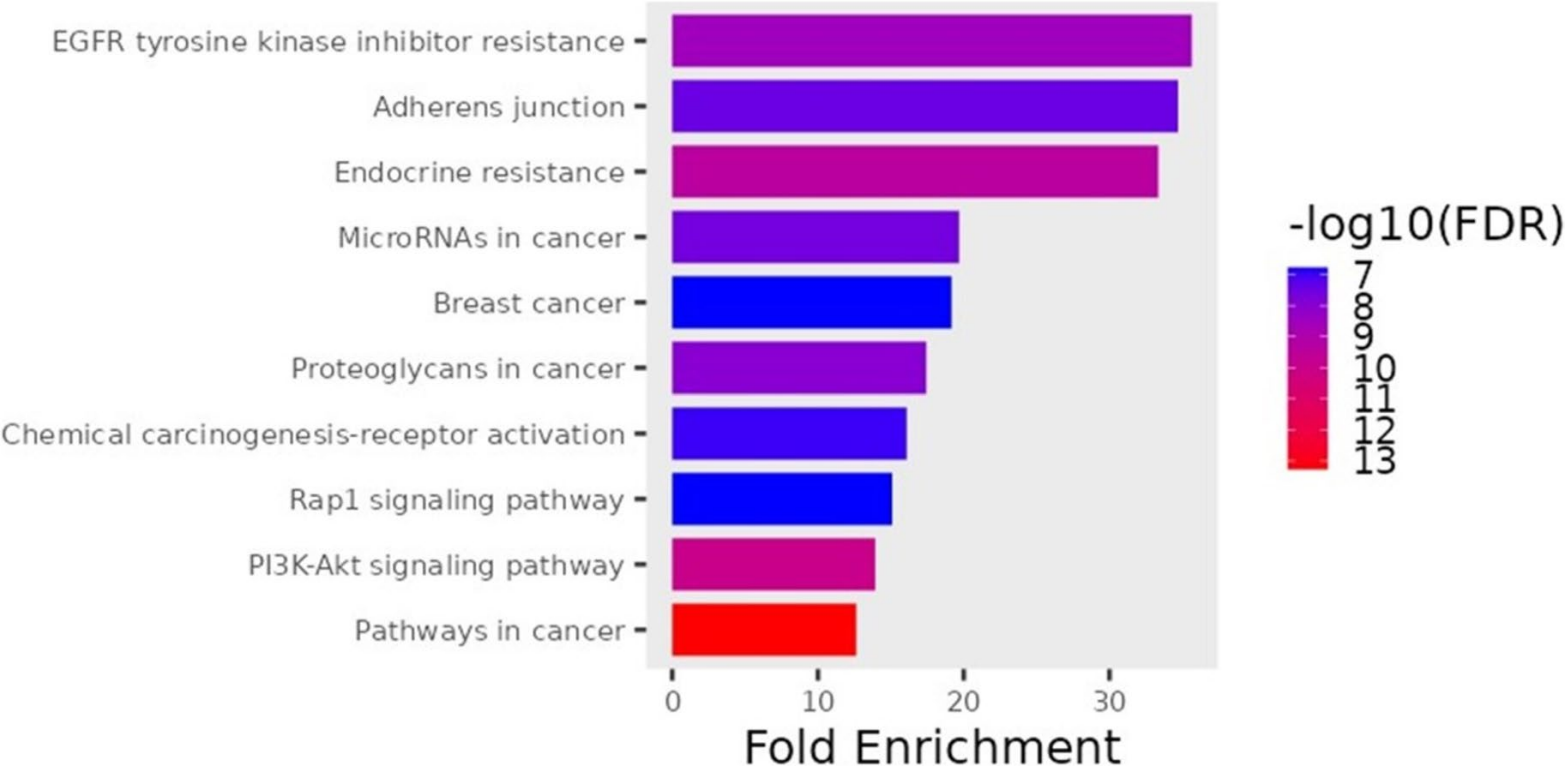
Pathway enrichment analysis of common bioactive compounds and drug therapeutic target genes in tongue cancer identified the top 10 significant pathways. The names of KEGG signal pathways are represented on the ordinate, whereas the proportion of genes is depicted on the abscissa. The degree of enrichment and significance were arranged in descending order from top to bottom

**Fig. 15:**
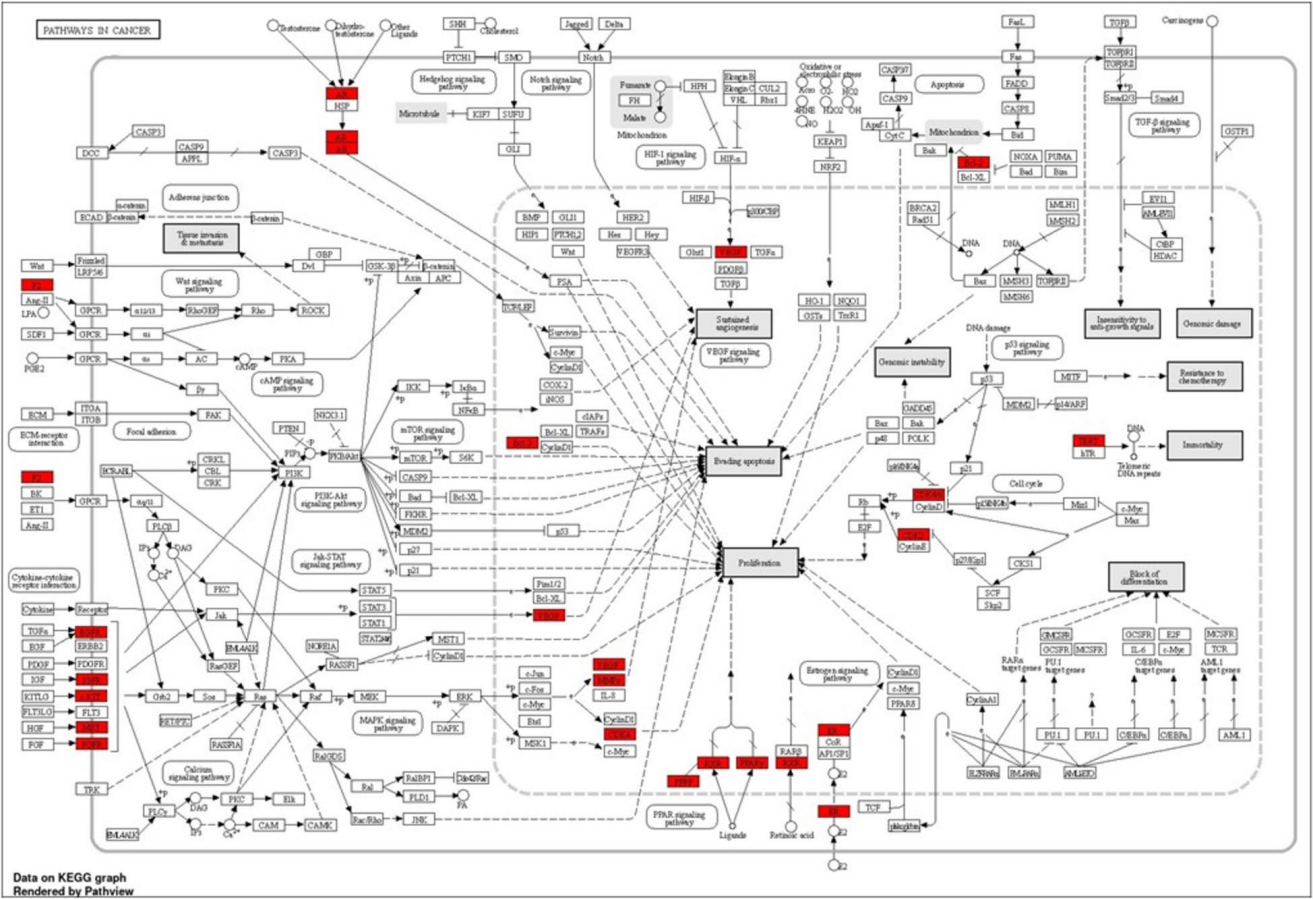
The Shiny GO database provides a diagrammatic representation of pathways and genes associated with cancer treatment. The red rectangles in the diagram represent the hit genes involved in tongue cancer treatment

Through KEGG pathway enrichment analyses, we identified the metabolic pathways associated with potential therapeutic targets for tongue cancer treatment., In the generated bar graph (Fig. 14), the top 10 signaling pathways are presented, arranged in ascending order based on their P-values from smallest to largest. The results showed that pathways that are enriched with hub genes are pathways in cancer, PI3K-Akt signaling pathway, MicroRNAs in cancer, EGFR tyrosine kinase inhibitor resistance, and Proteoglycans in cancer (Table 4). A diagrammatic representation of pathways and genes involved in cancer treatment was formed by the Shiny GO database. Red rectangles represent the hit genes involved in the treatment of tongue cancer (TERT, Bcl-2, CDK4/6, CDK2, VEGF, ER, RXR,c-KIT, MET, FGFR, PPAR8, PFFP, MMPs, CDK4, AR, F2, IGFR). These hit genes are targeted by both Rumex bioactive compounds and cisplatin (Figs. 14 and 15).

**Table 4:** Top 10 KEGG pathways involved in pathogenesis and treatment of tongue cancer

Pathway	Genes
EGFR tyrosine kinase inhibitor resistance	EGFR IGF1R KDR MET AXL BCL2 SRC VEGFA
Adherent junction	CSNK2A1 EGFR FGFR1 CSNK2A3 IGF1R MET SRC
Endocrine resistance	CDK4 EGFR ESR1 ESR2 IGF1R MMP2 MMP9 BCL2 SRC
MicroRNAs in cancer	CDK6 DNMT1 EGFR MCL1 MET MMP9 ABCB1 BCL2 VEGFA
Breast cancer	CDK4 CDK6 EGFR ESR1 ESR2 FGFR1 IGF1R KIT
Proteoglycans in cancer	EGFR ESR1 FGFR1 IGF1R KDR MET MMP2 MMP9 SRC VEGFA
Chemical carcinogenesis-receptor activation	CYP1A1 EGFR ESR1 ESR2 AR BCL2 RXRA SRC VEGFA
Rap1 signaling pathway	DRD2 EGFR FGFR1 IGF1R KDR KIT MET SRC VEGFA
PI3K-Akt signaling pathway	CDK2 CDK4 CDK6 EGFR FGFR1 IGF1R KDR KIT MCL1 MET NTRK2 BCL2 RXRA VEGFA
Pathways in cancer	CDK2 CDK4 CDK6 EGFR ESR1 ESR2 F2 FGFR1 IGF1R AR KIT MET MMP2 MMP9 PPARG BCL2 RXRA TERT VEGFA

### Bioactive − common target − drug-autophagy

**Fig. 16:**
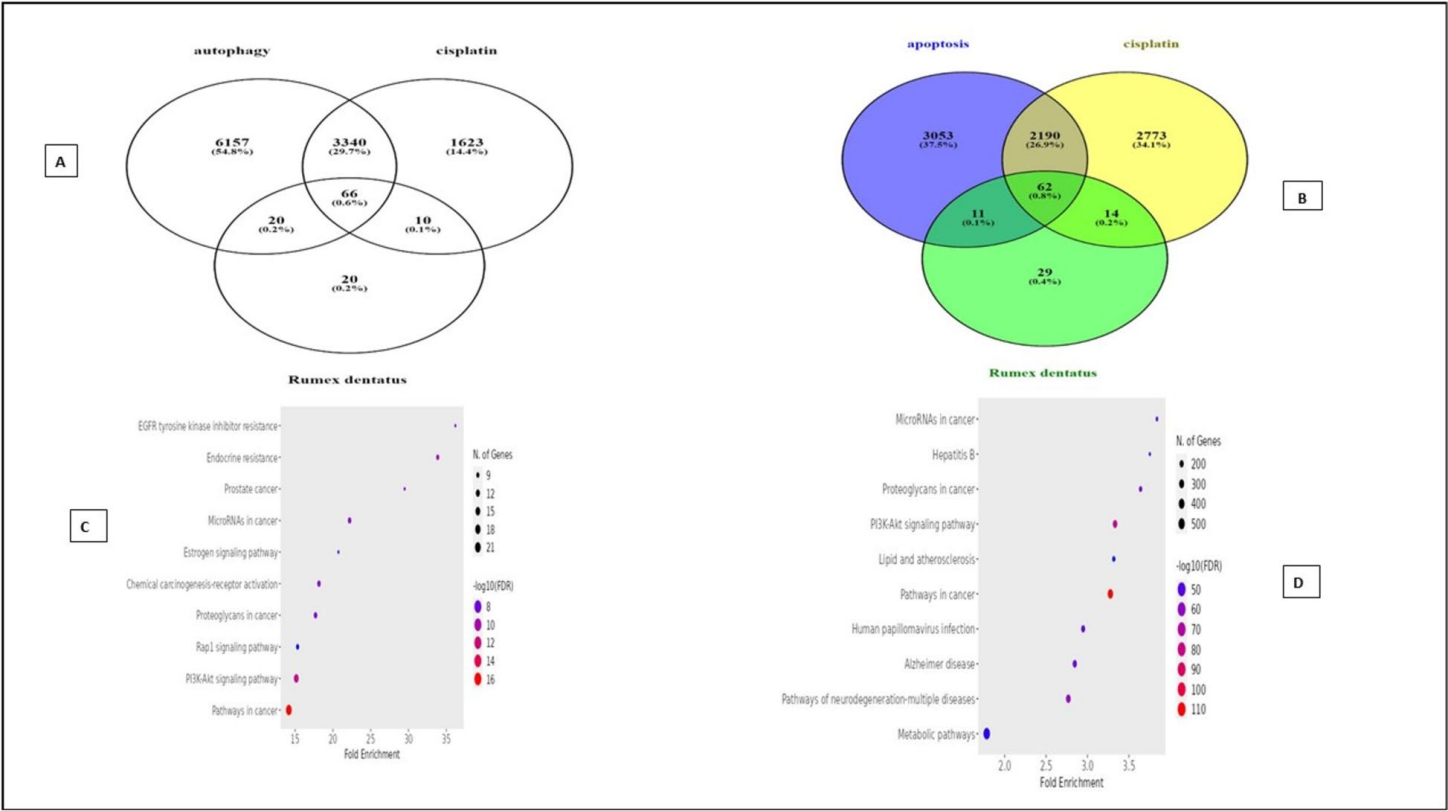
**A** The Venn diagram depicts the shared target genes of the bioactive compounds from *R. dentatus,* autophagy, and cisplatin. The overlapping area in the diagram indicates the common target genes. **B** The Venn diagram shows the target genes shared by bioactive compounds obtained from *R. dentatus*, apoptosis, and cisplatin. The overlapping areas in the diagram represent common target genes. **C** Bubble plot of the top 10 target genes associated with bioactive compounds, cisplatin, and autophagy. **D** A bubble chart displaying the top 10 target genes that are linked to bioactive compounds, cisplatin, and apoptosis

The common target genes among selected bioactive compounds, cisplatin, and autophagy were then identified through the intersection of their obtained targets, and a Venn diagram was generated for this purpose. 66 intersection targets were obtained (Fig. 16) (Table 5).

**Table 5:** Intersection of bioactive targets, cisplatin targets, autophagic targets, apoptosis targets

**Bioactive − common target − drug-autophagy**	**Bioactive − common target − drug-apopto**sis
EGFR	ABCB1
MET	ABCC1
TERT	ABCG2
KIT	AKR1C3
ESR1	ALOX12
AR	ALPL
CDK4	APEX1
BCL2	APP
PPARG	AR
VEGFA	AURKA
SRC	AXL
FGFR1	BCL2
MMP9	BRD4
AURKA	CA12
MMP2	CDK1
ABCB1	CDK2
CDK2	CDK4
PARP1	CDK5
KDR	CDK6
IGF1R	CSNK2A1
CYP19A1	CSNK2A3
ESR2	CYP19A1
DNMT1	CYP1A1
SERPINE1	DNMT1
CDK1	DPP4
CYP1A1	DRD2
APP	DYRK1A
ABCC1	EGFR
TOP2A	ELAVL3
MCL1	ESR1
CDK6	ESR2
F2	F2
APEX1	FGFR1
PKM	GLO1
RXRA	HSD17B1
ALOX5	IGF1R
RIPK1	KDR
CSNK2A1	KIT
NTRK2	LCK
MMP13	LIMK1
AURKB	MAOB
CDK5	MCL1
XBP1	MET
AXL	MMP12
PTGS1	MMP13
DRD2	MMP2
XDH	MMP9
HSP90AB1	NOX4
DPP4	NTRK2
BRD4	PARP1
PIM1	PKM
AKR1C3	PPARG
RAD52	RAD52
ALOX12	RIPK1
DYRK1A	RXRA
GLO1	SERPINE1
NOX4	SIRT5
CDK9	SRC
MAOB	TERT
CA12	TNKS
PGD	TNKS2
TNKS	TOP2A
TNKS2	TTR
SIRT5	VEGFA
PTPRS	XBP1
IP6K2	XDH

### Bioactive − common target − drug-apoptosis

The common target genes among selected bioactive compounds, cisplatin, and apoptosis were then identified through the intersection of their obtained targets, and a Venn diagram was generated for this purpose. 62 intersection targets were obtained (Fig. 16) (Table 4).

### Analysis of signaling pathways for bioactive compounds, drugs, and their role in inducing apoptosis or autophagy

The bubble plot in (Fig. 16) illustrates the top 10 KEGG pathways involved in autophagy and apoptosis in cancer respectively, where the bubble size and color serve as indicators of enrichment gene count and p-value, respectively. Colors from red to blue correspond to a smaller p-value, and a larger bubble size, indicates a greater abundance of enriched therapeutic genes within a specific pathway. This suggests a stronger association of the identified KEGG pathways with tongue cancer treatment compared to other terms. Our analysis highlighted that the two highly significant pathways involved in both apoptosis and autophagy are pathways in cancer and the PI3K-Akt signaling pathway. These two pathways involve many hub genes targeted by both cisplatin and bioactive compounds of *R. dentatus* L., and it is worth mentioning that these genes are involved in both pathogenesis and treatment of tongue cancer (Table 6). Those hub genes involved in pathways of cancer are Bcl2, EGFR, ESR1, MMP9, PPARG, and MMP2 and those involved in PI3K-Akt signaling pathways are Bcl2, EGFR, CDK2, and MCL1 these genes are considered therapeutic targets for tongue cancer.

**Table 6:** Top two KEGG pathways involved in apoptosis and involved genes

PI3K-Akt signaling pathway:
BCL2L11 CDK2 CDK4 CDK6 CDKN1A CDKN1B GNB5 YWHAQ CHUK COL1A1 COL1A2 COL2A1 COL4A1 COL4A3 COL4A4 COL4A5 COL6A1 COL6A2 COL6A3 COL9A1 COL9A2 COL9A3 COMP CREB1 CSF1 CSF1R CSF3 EFNA2 EFNA5 EGF EGFR EPHA2 EIF4B EIF4E EIF4EBP1 EPO EPOR ERBB2 ERBB3 ERBB4 AKT1 AKT2 FGF1 FGF2 FGF3 FGF4 FGF5 FGF6 FGF7 FGF8 FGF9 FGF10 FGFR1 FGFR3 FGFR2 FGFR4 VEGFD FOXO3 FLT1 FLT3 FLT4 FN1 PIK3R5 MTOR FGF20 GHR FGF22 GNB3 ANGPT1 ANGPT2 GRB2 GSK3B HGF NR4A1 HSP90AA1 HSP90AB1 TNC IBSP IFNA1 IFNA2 IFNA4 IFNA5 IFNA6 IFNA7 IFNA8 IFNA10 IFNA13 IFNA14 IFNA16 IFNA17 IFNA21 IFNAR1 IFNAR2 IFNB1 IGF1 IGF1R IGF2 IKBKB IL2 IL2RA FASLG IL2RG IL3 IL4 IL4R IL6 IL6R IL7 INS INSR IRS1 ITGA2 ITGA2B ITGA3 ITGA4 ITGA7 ITGAV ITGB1 ITGB3 ITGB4 ITGB5 ITGB6 JAK1 JAK2 JAK3 AREG KDR KIT KRAS LAMA2 LAMA3 LAMA5 LAMB3 LAMC2 MCL1 MDM2 MET MYC NFKB1 NGF NGFR NOS3 NRAS NTF3 NTF4 NTRK1 NTRK2 PDGFA PDGFB PDGFRA PDGFRB PDPK1 PGF PIK3CA PIK3CB PIK3CD PIK3CG PIK3R1 PIK3R2 DDIT4 PPP2CA PPP2R1A PPP2R1B PPP2R2A PPP2R2B PRKAA1 PRKAA2 PRKCA PKN2 MAPK1 MAPK3 MAP2K1 MAP2K2 PRL PRLR RELN PTK2 RPTOR RAC1 RAF1 RBL2 GNB4 CCND1 BCL2 RELA BCL2L1 RHEB RPS6 RPS6KB1 RPS6KB2 RXRA BDNF MLST8 SGK1 SOS1 SOS2 SPP1 BRCA1 STK11 SYK TEK TGFA THBS1 THBS3 THBS4 TLR2 TLR4 TNR TP53 TSC1 TSC2 VEGFA VEGFB VEGFC VTN VWF YWHAZ PDGFD FGF23 CASP9 ITGA10 IKBKG FGF18 FGF17 FGF16 CCND2 CCND3 CREB3L1 G6PC3 CD19 MAGI2
**Pathways in cancer**
ADCY3 ADCY5 RASSF1 CHUK ADCY9 CKS1B CKS2 COL4A1 COL4A3 COL4A4 COL4A5 CREBBP CRKL CSF1R IL23R CTNNA1 CTNNB1 DAPK1 CALML6 DDB2 GADD45A NQO1 JAG1 AGT AGTR1 DVL1 DVL2 DVL3 E2F1 E2F3 EDN1 EDNRA EDNRB EGF EGFR EP300 EPAS1 EPO EPOR ERBB2 AKT1 AKT2 ESR1 ESR2 ETS1 MECOM F2 FGF1 FGF2 FGF3 FGF4 FGF5 FGF6 FGF7 FGF8 FGF9 FGF10 FGFR1 FGFR3 FGFR2 FGFR4 FH VEGFD FOXO1 FLT3 PLCB1 FLT4 FN1 FOS ALK MTOR ABL1 FZD2 APPL1 FGF20 FGF22 GLI1 GLI2 GLI3 GNA11 GNAI2 GNAI3 GNAQ GNAS GNB3 GRB2 CTNNA3 GSK3B GSTM1 GSTM2 GSTP1 MSH6 HDAC1 HDAC2 HGF HIF1A HMOX1 APAF1 APC BIRC2 BIRC3 XIAP BIRC5 HSP90AA1 HSP90AB1 IFNA1 IFNA2 IFNA4 IFNA5 IFNA6 IFNA7 IFNA8 IFNA10 IFNA13 IFNA14 IFNA16 IFNA17 IFNA21 IFNAR1 IFNAR2 IFNG IFNGR1 IGF1 IGF1R IGF2 FAS IKBKB IL2 IL2RA FASLG IL2RG IL3 IL4 IL4R IL5 IL6 IL6R IL6ST IL7 CXCL8 IL12A IL12RB1 IL12RB2 IL13 IL15 AR ITGA2 ITGA2B ITGA3 ITGAV ITGB1 ARAF JAG2 JAK1 JAK2 JAK3 JUN JUP KIF7 KIT KNG1 KRAS RHOA LAMA2 LAMA3 LAMA5 LAMB3 LAMC2 LRP5 ARNT SMAD2 SMAD3 SMAD4 MAX MDM2 MET MITF MLH1 MMP1 MMP2 MMP9 MSH2 MSH3 MYC NFE2L2 NFKB1 NFKB2 NFKBIA NOS2 NOTCH1 NOTCH2 NOTCH3 NRAS NTRK1 LEF1 POLK PDGFA PDGFB PDGFRA IL23A PDGFRB SUFU CALML5 PGF PIK3CA PIK3CB PIK3CD PIK3R1 PIK3R2 PLCB2 PLCB3 PLCB4 PLCG1 PLCG2 CYCS DLL4 PPARG PRKACB PRKACG PRKCA PRKCG MAPK1 MAPK3 MAPK8 MAP2K1 MAP2K2 PTCH1 PTGS2 PLEKHG5 PTK2 BAK1 BAX RAC1 RAC3 RAD51 RAF1 RALGDS RARA RARB RB1 GNB4 CCND1 BCL2 RELA RET BCL2L1 ROCK1 BCR RPS6KB1 RPS6KB2 BDKRB1 BDKRB2 RXRA CXCL12 SHH BMP2 SKP2 SLC2A1 BMP4 SMO SOS1 SOS2 SP1 BRAF BRCA2 STAT1 STAT3 STAT4 STAT5B STAT6 TCF7L2 TERT TGFA TGFB1 TGFB2 TGFB3 TGFBR1 TGFBR2 TP53 TPM3 TPR TRAF3 TRAF6 VEGFA VEGFB VEGFC VHL WNT1 WNT5A WNT6 WNT7A WNT10B WNT11 ZBTB16 PAX8 CXCR4 FZD5 CCDC6 NCOA4 WNT10A FGF23 CALM3 CALML3 CAMK2A CAMK2B NCOA3 FZD4 FZD7 CASP3 CASP8 CASP9 IKBKG RUNX1 PTCH2 FADD FGF18 FGF17 FGF16 CCNA2 CCNA1 CCND2 CCND3 WNT3A RPS6KA5 ROCK2 TRAF4 KEAP1 RBX1 CDC42

### Drug-likeness and ADME properties

The drug likeness score of the three selected bioactive compounds was evaluated using Lipinski's rule of five The rule states that an orally active drug-like compound should not have more than one violation of the following criteria as stated by Lipinski's rule: hydrogen bond donors not greater than 5, hydrogen bond acceptors not greater than 10, molecular weight not greater than 500 Da, and octanol–water partition coefficient (log P) not greater. According to this rule, we could say that the three selected bioactive compounds emodin, quercetin, and taxifolin will have drug-like properties. They also have high oral absorption according to the data obtained from the SwissADME database (Table 7).

**Table 7:** Prediction of Drug-likeness and ADME Properties of Bioactive Compounds

Pharmacokinetics	Emodin	Quercetin	Taxifolin
GI absorption	High	High	High
BBB permeant	No	No	No
P-gp substrate	No	No	No
CYP1A2 inhibitor	Yes	Yes	Yes
Drug likeness	Yes; 0 violation	Yes; 0 violation	Yes; 0 violation

## Discussion

Oral cavity malignancies are the most prevalent among head and neck cancers and rank as the sixth most common cancer globally. Treatment typically involves surgery, radiotherapy, and chemotherapy, with cisplatin being a frequently utilized chemotherapeutic agent. Cisplatin functions by inducing DNA damage to inhibit cell proliferation and trigger apoptosis in cancer cells. However, its efficacy is often limited by the development of resistance, leading to relapse [6, 8, 30, 31].

Alternative or complementary therapies are being investigated to address cisplatin resistance, including the utilization of natural products to enhance cancer cell chemosensitivity. Herbal-based treatments, such as those derived from the Rumex genus, have demonstrated potential in this area. Rumex dentatus in particular has exhibited significant efficacy against various cancer cell lines [32].

The genus Rumex, belonging to the Polygonaceae family, exhibits a significant global distribution with approximately 200 known species within this genus, many of which demonstrate pharmacological effects. R. dentatus L. is particularly noteworthy for its efficacy. It has also been successfully evaluated against various cancer cell lines in research studies. While the roots of this plant have been extensively studied and screened, limited research has been conducted on the aerial part chemistry and biology, especially in the third stage of growth. Consequently, the aerial part of R. dentatus L. was selected in the third stage of growth to investigate its phenolic content and its in vitro potential synergistic effect when combined with cisplatin on tongue carcinoma cell line.

In this study, the phenolic aglycone profile of the aerial part of R. dentatus L. in the third growth stage was investigated for the first time using UPLC-ESI-MS/MS analysis. The extract comprises flavanol (quercetin, kaempferol), methylated flavanol (isorhamnetin, kaempferide), flavone (luteolin), flavanone (eriodictyol), and flavanol (taxifolin) compounds, which are phenolic structures of the flavonoid type. Additionally, the anthraquinone compounds detected were exclusively emodin and alo-emodin. These classes of compounds are well-established as anticancer agents.

The objective of the present study is to evaluate the in vitro potential positive impact of combining cisplatin with Rumex dentatus extract on the tongue carcinoma cell line. Our hypothesis posits that Rumex dentatus extract will enhance the anticancer efficacy of cisplatin by suppressing cell proliferation, inducing cell cycle arrest, facilitating programmed cell death, and inhibiting autophagy.

Network pharmacology was utilized to forecast the potential bioactive compounds of Rumex dentatus extract, their respective target genes, and the signaling pathways influenced by these bioactive compounds in the context of tongue carcinoma.

Our results revealed that a concentration-dependent inhibitory effect of *R. dentatus* extract and cisplatin on HNO-97 tongue carcinoma cells was observed when each was administered independently. The IC50 values for Rumex dentatus and cisplatin were 97.9 μg/mL and 0.328 μg/mL, respectively, indicating moderate cytotoxicity for Rumex dentatus. Rumex dentatus exhibited weak cytotoxicity against normal oral epithelial cells, suggesting its relative safety. Flow cytometry analysis revealed that Rumex dentatus significantly increased the sub-G1 population (indicative of apoptosis) in HNO-97 cells, with even higher levels observed when combined with cisplatin. The combination treatment significantly increased the expression of pro-apoptotic p53 and decreased anti-apoptotic BCL2 expression. Rumex dentatus also reduced the expression of autophagy protein ATG7, with greater reductions observed in the combination treatment.

Both Rumex dentatus and cisplatin exhibited dose-dependent inhibition of the HNO-97 cells. The IC50 value for Rumex dentatus was 97.9 µg/mL, which categorizes it as a moderately active agent, according to the classification by Atjanasuppat et al. This finding supports the hypothesis that Rumex dentatus, although less potent than cisplatin, demonstrates significant cytotoxic activity against tongue carcinoma cells. The moderate antiproliferative activity of Rumex dentatus suggests that while it is not as efficacious as cisplatin, it exerts a measurable impact on inhibiting cancer cell growth, rendering it a potential candidate for combination therapy to enhance cisplatin's effects. This combination approach holds the potential to decrease the required therapeutic concentration of cisplatin, thereby mitigating its associated side effects, while still preserving the intended therapeutic response.

In the current study Rumex dentatus was evaluated against the OEC normal oral epithelial cell line, demonstrating an IC50 > 100 µg/mL, which indicates minimal cytotoxicity against normal cells. This observation suggests a favorable safety profile for Rumex dentatus, as it exhibits selective toxicity against cancer cells while demonstrating limited effects on normal cells. This finding is significant, as a primary challenge in chemotherapy is achieving selective toxicity. The minimal effect of Rumex dentatus on normal cells, coupled with its moderate activity on cancer cells, underscores its potential as a safer adjunct to conventional chemotherapy, such as cisplatin, which is associated with severe side effects.

To our knowledge, it is the first study to investigate the synergistic effect of *R. dentatus* L. in association with cisplatin on tongue carcinoma.

Nevertheless, recent investigations have highlighted the anticancer properties of the leaves of *R. dentatus* L. alone and its ability to inhibit the proliferation of the MDA-MB-231 breast cancer cell line. Additionally, it has been documented to induce apoptosis and suppress cell survival in the MDA-MB-231 breast cancer cell line [33]. Flavonoids such as quercetin and taxifolin, as well as anthraquinones such as emodin, have been extensively documented for their anticancer properties, including the induction of apoptosis, cell cycle arrest, and inhibition of cancer cell proliferation in both prostate and breast cancer cells [34].

Flow cytometric analysis was employed to determine if Rumex enhances cisplatin's antiproliferative activity by disrupting cell division. The cell cycle phases include G1, S, G2, and mitosis (M). In G1, cell size increases, and RNA and proteins are produced for DNA synthesis. DNA replicates during the S phase, and new proteins are synthesized in G2. Nuclear and cytoplasmic divisions occur in the M phase. The reported antitumor efficacy of cisplatin has been closely associated with its capacity for DNA adduct formation, leading to cell-cycle arrest in numerous cancer cells [11].

The results of our study revealed that the untreated HNO-97 cells exhibited a normal cell cycle distribution, with low levels of cells in the sub-G1 phase, which is associated with apoptotic cell death. Normal cells maintained normal proliferation with low spontaneous apoptosis. Treatment with Rumex dentatus resulted in a significant increase in the sub-G1 cell population, indicating that the extract induces apoptotic cell death. This means that Rumex dentatus alone exhibits a pronounced pro-apoptotic effect on the HNO-97 cells, as evidenced by the accumulation of cells in the sub-G1 phase, this observation confirms its cytotoxic activity. The observed decrease in the G0/G1, S, and G2/M phases suggests that fewer cells are progressing through these stages of the cell cycle, as many are being directed toward apoptosis. This finding indicates that the mechanism of action of Rumex dentatus involves the induction of apoptosis, thereby inhibiting the proliferation of cancer cells.

The combination of Rumex dentatus and cisplatin resulted in a significant increase in the sub-G1 cell population compared to both the control and cisplatin alone, indicating enhanced apoptosis. This effect also led to further reductions in the number of cells in the G0/G1, S, and G2/M phases, as more cells were directed toward cell death rather than cell cycle progression.

Previous studies revealed that the methanol and chloroform extracts of *R. dentatus* may have anti-cancer compounds that are potentially useful in the treatment of human breast cancer [35]. Emodin was reported to induce cytotoxicity in SW480 and SW620 colorectal cancer cells, but not to a similar extent in normal human colon CCD 841 [36, 37].

The synergistic increase in the sub-G1 population when Rumex dentatus is combined with cisplatin is a crucial finding. It demonstrates that the combination treatment induces greater apoptosis than either agent alone, supporting the earlier conclusion that the combination exhibits a synergistic effect in cancer cell elimination. This observation is consistent with the Combination Index (CI) calculation, which suggested synergy between these two agents.

The B-cell lymphoma 2 (BCL-2) protein family consists of key regulators that control both pro-apoptotic and anti-apoptotic activities. Anti-apoptotic proteins, such as BCL-2 and BCL-XL, inhibit apoptosis and promote cell survival. Overexpression of BCL-2 enhances cell survival and proliferation, and recent advancements have led to the development of inhibitors targeting BCL2.

Additionally, p53, a nuclear transcription factor, plays a critical role in inducing cell cycle arrest and apoptosis. In many cancers, the p53 pathway is deactivated. Promising anticancer therapies aim to restore p53 function through gene therapy, RNA interference, and small molecules designed to target p53 [12, 14].

About 50% of known cancers exhibit p53 inactivation, and p53 induces apoptosis by activating the Bax gene, a critical member of the Bcl-2 family. Therefore, targeting Bcl-2 through the p53 pathway represents an effective strategy for combating cancer [38, 39]. Accordingly, our study tried to explore whether the apoptotic effect of *Rumex dentatus* could be achieved through the inhibition of Bcl-2.

Treatment with the combination of Rumex dentatus and cisplatin significantly upregulated the gene expression of the p53 protein. As a tumour suppressor, p53 plays a crucial role in inducing apoptosis by initiating the transcription of genes involved in cell cycle arrest and programmed cell death. The combination treatment also significantly downregulated BCL2 gene expression. BCL2, an anti-apoptotic protein that promotes cellular survival and proliferation through the inhibition of apoptotic signals, exhibits reduced expression, which is crucial for enhancing apoptosis in neoplastic cells.

These molecular alterations indicate that the combination of Rumex dentatus and cisplatin functions synergistically to induce apoptosis in tongue carcinoma cells. Through the concurrent upregulation of pro-apoptotic signals (via p53) and downregulation of anti-apoptotic defences (via BCL2), the combination enhances the efficacy of cisplatin, potentially overcoming resistance mechanisms and improving therapeutic outcomes. This suggests the potential of Rumex dentatus as a valuable adjuvant therapy to augment the apoptotic effect of cisplatin in the treatment of tongue cancer, which aligns with the research objective of identifying methods to enhance cisplatin's efficacy.

Studies showed that Rumex dentatus Inhibits Cell Proliferation, Arrests Cell Cycle, and Induces Apoptosis in MDA-MB-231 Cells through Suppression of the NF-κB Pathway and its subsequent transcripts, Bcl-xl, Bcl-2, Cyclin D1, survivin, and XIAP.

Studies have demonstrated that the beneficial impact of combining cisplatin with phytochemicals in the treatment of various cancer types including bladder cancer and gastric cancer [33, 40].

Autophagy is a cellular process that maintains homeostasis through the degradation of damaged components. In cancer, it exhibits a dual role: tumor suppression in normal cells and survival support in established tumors. ATG7, a key protein in autophagosome formation, plays a crucial role in cancer. Inhibition of ATG7 can prevent the development of precancerous lesions, and increased resistance to certain therapies. Thus, ATG7 is of significant importance in both cancer progression and treatment responses [41–46].

The findings of our study regarding the effects of Rumex dentatus extract on autophagy protein ATG7 expression in HNO-97 cells provide significant insights into the potential mechanisms underlying its therapeutic effects. A substantial reduction in ATG7 gene expression following treatment with Rumex dentatus extract suggests that the extract may inhibit autophagy in tongue carcinoma cells. This observation aligns with the broader understanding of autophagy's role in cancer, wherein autophagy can function as a survival mechanism in established tumors. By decreasing ATG7 expression, Rumex dentatus may disrupt this survival pathway, potentially increasing the vulnerability of cancer cells to apoptosis or other death signals. Previous investigations demonstrated that emodin inhibited cell metastasis in hepatocellular carcinoma (HCC) through the interplay between autophagy and epithelial-mesenchymal transition (EMT) [47]. On the other hand, quercetin-inhibition of autophagy contributes to apoptosis in A549 and H1299 lung cancer cells, which involves the SIRT1/AMPK signaling pathway. Quercetin has been shown to inhibit cancer through the modulation of apoptosis and autophagy through the targeting of the PI3K/Akt/mTOR, Wnt/-catenin, and MAPK pathways [18, 48].

Furthermore, when Rumex dentatus was combined with cisplatin, the decrease in ATG7 gene expression was more pronounced compared to either treatment alone. This observation suggests a synergistic interaction between Rumex dentatus and cisplatin in inhibiting autophagy. Given that cisplatin is known for inducing DNA damage and apoptosis, the combination may enhance the overall cytotoxic effects by simultaneously inhibiting a key survival mechanism (autophagy) and promoting cell death.

In this study, we employed network pharmacology to anticipate the mechanism responsible for the therapeutic benefits of *R. dentatus* L. in the treatment of tongue carcinoma in correlation to the results of the in vitro investigation. Through our analysis, we pinpointed and scrutinized 3 active antitumor compounds, taxifolin, quercetin, and emodin which constitute essential bioactive compounds contributing to the anti-tumor activities of *R. dentatus* L.

Taxifolin and emodin, phenolic compounds, have attracted significant interest due to their extensive pharmacological effects. They also exhibit anticancer properties. Taxifolin showed inhibition of angiogenesis, cytochrome P450 enzymes, P-glycoprotein, reactive oxidative species (ROS), and modulation of cell cycle regulators. Additionally, taxifolin is implicated in inducing apoptosis [45]. Studies have unveiled that quercetin effectively hinders the proliferation of a broad spectrum of cancer cell lines, demonstrating its capability to induce apoptosis and/or arrest the cell cycle [46].

Emodin, a natural anthraquinone derivative present in the roots and rhizomes of various plants, has been the subject to pharmacological studies that highlight its diverse biological functions [30]. Emodin showed anticancer activity, as evidenced by studies illustrating its ability to impede cell growth in multiple cancer cell types. Furthermore, it modulates genes associated with the regulation of cell apoptosis, oncogenesis, cell proliferation, as well as the invasion and metastasis of cancer cells [49].

The results of our network study showed that the three selected bioactive compounds targeted a total of 154 human genes. Venny shape revealed that there are 66 common target genes for Rumex, cisplatin, and tongue carcinoma. The PPI network comprising 66 intersection genes was established. Additionally, through the utilization of CytoHubba, it has been identified that PARP1, CDK2, MCL1, ESR1, MMP2, SRC, EGFR, PPARG, BCL2, and MMP9 could potentially serve as key target genes for bioactive compounds in the treatment of tongue carcinoma. Among those hub genes ESR1, EGFR, and BCL2 are deemed core targets within the PPI network as determined by network topology parameters. Research findings suggest a potential association between ESR1 and the risk of late-onset prostate cancer [50]. A thorough examination of the current literature on EGFR and cancer prognosis highlights a consistent correlation between elevated EGFR levels and unfavorable patient outcomes across various cancer types, including head and neck, ovarian, cervical, bladder, and esophageal cancer [51]. The elevated expression of the Bcl-2 protein in tumor cells, compared to normal cells, indicates that inhibitors targeting this protein have minimal impact on normal cells. Thus, a promising therapeutic approach for overcoming tumor cell resistance to apoptosis involves inhibiting the anti-apoptotic Bcl2 protein, aligning with novel strategies involved in tumor pathogenesis [52]

Gene ontology is a bioinformatics tool used to categorize genes based on their functions and roles in biological processes. GO analyses would have been employed to categorize the genes affected by bioactive compounds and identify the biological processes and pathways influenced by these compounds in the treatment of tongue carcinoma.

The findings from the GO analyses indicate that the bioactive compounds can modulate different biological processes and pathways. Our analysis highlighted that the principal enriched Biological Process (BP) categories included are GO:1,901,700: Response to oxygen-containing compound, GO:0043067: Regulation of programmed cell death, GO:0010035: Response to inorganic substance, GO:0008284: Positive regulation of cell population proliferation, GO:0006468: Protein phosphorylation, GO:0010647: Positive regulation of cell communication, GO:0023056: Positive regulation of signaling, GO:0016310: Phosphorylation. This modulation may involve influencing specific cellular activities, signaling pathways, or molecular interactions that play a crucial role in the development or progression of tongue carcinoma. The analysis of cellular components (CC) revealed that GO:0000307 cyclin-dependent protein kinase holoenzyme complex. GO:0043073 germ cell nucleus. GO:1,902,911 protein kinase complex, GO:0000781 chromosome telomeric region, GO:0043235 receptor complex, GO:0045121 membrane raft, GO:0098857 membrane microdomain, GO:0098687 chromosomal region, O:0005667 transcription regulator complex GO:0005739 mitochondrion.

KEGG pathway analysis was conducted to explore the interactions among target genes involved in tongue carcinoma. The results of the KEGG analysis revealed the specific pathways through which the target genes can exert their effects in the pathogenesis and treatment of tongue carcinoma. This analysis provides valuable insights into the molecular mechanisms and signaling cascades that contribute to the observed therapeutic actions of bioactive compounds against tongue carcinoma.

The KEGG analysis indicated the potential pathways through which bioactive compounds may exert therapeutic effects on tongue carcinoma are PI3K-Akt signaling pathway, MicroRNAs in cancer, EGFR tyrosine kinase inhibitor resistance and proteoglycans in cancer.

The PI3K-AKT signaling pathway is a key pathway for cancer therapy and is involved in various biological processes such as apoptosis, cell proliferation, and cell cycle [53]. The synergy between the PI3K-AKT pathway and various chemotherapeutic agents, including doxorubicin, etoposide, topotecan, cisplatin, vincristine, and taxol, is well-documented, leading to heightened tumor sensitivity to chemotherapy. Notably, inhibiting PI3K-AKT has been shown to trigger apoptosis and impede tumor growth in primary neuroblastoma cells derived from patients and in an in vivo neuroblastoma model. Furthermore, early clinical investigations have indicated that the combination of PI3K-AKT inhibition with chemotherapy is both safe and well-tolerated [54].

Data from several studies presented the impact of PI3K/AKT pathway dysregulation on the survival of SCC patients originating from different parts of the body, such as the cervix, oral cavity, head and neck, and skin. Furthermore, targeted therapies against this pathway have shown effectiveness in reducing tumor burden in both animal models and clinical settings. Lastly, several molecules that regulate the PI3K/AKT pathway can serve as diagnostic markers for different types of SCCs. The PI3K-Akt pathway also stimulates tumor angiogenesis by upregulating vascular endothelial growth factor (VEGF). The activation of the PI3K-Akt pathway has been shown to enhance the motility and invasiveness of oral squamous cell carcinoma (OSCC) cells. This effect is attributed to the regulation of proteins involved in cytoskeletal rearrangement, matrix degradation, and epithelial-mesenchymal transition (EMT), ultimately facilitating the invasion of surrounding tissues and metastasis by cancer cells [13, 22, 29].

In 2007, the correlation between MicroRNAs and the metastasis process was initially unveiled when Li Ma, Robert Weinberg, and their colleagues investigated MicroRNAs expression profiles in various breast cancer cells, distinguishing between metastatic and non-metastatic cells, alongside healthy human mammary epithelial cells. This investigation led to the identification of several MicroRNAs associated with metastasis. Among them, MicroRNAs −101 demonstrated its efficacy in hindering the progression of oral cancer by suppressing migration and invasion [47].

MicroRNAs (miRNAs) have become important prognostic biomarkers and potential therapeutic targets in oral cancer. Research indicates that specific miRNAs are associated with clinical stage, metastasis, and patient survival, suggesting their potential as indicators for disease progression and prognosis. miR-31-5p promotes OSCC cell migration and invasion, while high expression of miR-99a is linked to a better prognosis. Conversely, overexpression of miR-183 predicts poor outcomes [55].

Several miRNAs are implicated in oral cancer cell proliferation, apoptosis, and metastasis. For example, miR-155 promotes cell proliferation, whereas miR-34a-5p and miR-204-5p inhibit metastasis. Therapeutically, miRNAs like miR-375 and miR-494-3p enhance radio sensitivity, while others such as miR-23a-3p and miR-1254 inhibit cancer cell proliferation and aggressiveness [56].

Inhibitors of the epidermal growth factor receptor tyrosine kinase (EGFR-TKI), has gained widespread acceptance for the treatment of metastatic EGFR-mutant non-small cell lung cancer (NSCLC), leading to notable improvements in outcomes. In head and neck squamous cell carcinoma (HNSCC), where EGFR is overexpressed in over 90% of cases, the use of EGFR-TKI has shown promising results. The findings underscore a distinct positive response of tongue cancer to EGFR-TKI indicating its potential utility in the management of this specific type of cancer.

EGFR tyrosine kinase inhibitor (TKI) resistance in oral squamous cell carcinoma (OSCC) can arise from various mechanisms. These may involve the activation of alternative signaling pathways (such as MET, HER2, IGF-1R), epithelial-mesenchymal transition (EMT), mutations in downstream signaling pathways (such as PI3K/AKT), increased glycolytic activity (via PKM2, GLUT1), and drug efflux mechanisms. The tumor microenvironment also plays a role by providing support for alternative growth signals. To combat this resistance, strategies could involve combination therapies targeting alternative pathways, glycolysis inhibitors (such as quercetin), and combining TKIs with immune checkpoint inhibitors.

Proteoglycans, a class of high-molecular-weight glycoproteins, are prominently present in the extracellular matrix of connective tissue, providing structural support to the body. They constitute a significant portion of the extracellular matrix, filling intercellular spaces [57]. In the intricate process of tumor angiogenesis, various proteoglycans play a role in influencing cell growth by interacting with growth factors, thus regulating cancer cell proliferation. Unlike other body tissues, the extracellular matrix (ECM) stands out as a vital component of connective tissue [58]. Proteoglycans are intricately linked to every stage of the metastatic cascade. Notably, specific proteoglycans seem to actively participate in various aspects of cancer progression, underscoring their potential as key players on the cancer cell surface [59]. The most significant signaling pathway affected in primary OSCC was found to be "proteoglycans in cancer." These findings could potentially improve the prognosis for patients with early-stage OSCC and lead to more effective therapeutic strategies. Gene set enrichment analysis was conducted to illustrate the important pathways and GO annotations impacted in primary OSCC. This analysis utilized genes associated with prominent clusters in the PPI network related to the etiology of the disease. The key pathway identified through this analysis was "proteoglycans in cancer".

It is worth saying that the previously mentioned pathways are enriched with hub genes that serve as key target genes for bioactive compounds in the treatment of tongue carcinoma.

To verify the in vitro study that revealed that autophagy and induction of cancer cell apoptosis are major mechanisms involved in the inhibition of tongue carcinoma cell proliferation induced by the Rumex and cisplatin combination. We carried out KEGEG analysis for Bioactive − common target-drug-autophagy and Bioactive − common target-drug-apoptosis. Results showed that pathways involved in both processes are enriched with hub genes that act as major targets involved in the treatment of tongue carcinoma.

Previous studies indicated that plant-derived compounds could emerge as favorable candidates for drug discovery after undergoing evaluations using drug-likeness filters [60]. Drug likeness is a concept that indicates the similarity between bioactive compounds and known drugs.

Our results revealed that the three bioactive compounds taxifolin, quercetin, and emodin have drug-like properties. They also have high oral absorption and bioavailability.

In summary, Rumex dentatus and cisplatin inhibited HNO-97 cell growth dose-dependently, with Rumex dentatus showing moderate antiproliferative activity and low cytotoxicity against normal cells indicating a favorable safety profile. Their combination had synergistic effects. Rumex dentatus induced apoptotic cell death by increasing the sub-G1 phase in HNO-97 cells. The combination treatment notably enhanced apoptosis compared to either agent alone, as shown by flow cytometry. Rumex dentatus, alone or with cisplatin, significantly reduced autophagy protein ATG7 and anti-apoptotic BCL2 expression, while increasing pro-apoptotic p53 expression in HNO-97 cells. Three key bioactive compounds in Rumex dentatus (quercetin, taxifolin, and emodin) target 66 common genes linked with cisplatin and tongue cancer, including EGFR, BCL2, and ESR1 as the most important genes identified. Pathways related to OSCC were identified by using GO and KEGG pathway analyses The three bioactive compounds comply with Lipinski's rule of five, indicating good drug-like properties and high oral absorption potential. So it could be concluded that Rumex dentatus shows promise in enhancing cisplatin's anticancer effects, suggesting its potential as an adjunctive therapy for tongue cancer.

The importance of this study arises from its ability to prove that the combination of Rumex dentatus extract and cisplatin demonstrates significant therapeutic potential for the treatment of tongue cancer. The extract enhances cisplatin's anticancer effects through the induction of apoptosis, disruption of the cell cycle, and inhibition of autophagy. Network pharmacology and gene expression analyses elucidate that Rumex targets key OSCC-related pathways, emphasizing its role in the treatment of this type of cancer. These findings provide a foundation for further preclinical and clinical investigations to explore the utilization of Rumex dentatus as an adjunctive therapy to existing chemotherapeutic agents, particularly for tongue carcinoma.

While the investigation demonstrates promising outcomes, particularly regarding the synergistic effect between Rumex dentatus and cisplatin, several limitations necessitate further research. These include the requirement for in vivo validation as in vivo studies are crucial for confirming and extending the results obtained in vitro. future studies will build on this work by incorporating in vivo experiments to validate the in vitro results, more extensive mechanistic studies, and a more comprehensive evaluation of safety, drug delivery mechanisms, and clinical applicability is also important in future research studies.

## Conclusion

We demonstrated that a combination of the extract of the aerial part of *R. dentatus* L and cisplatin significantly suppressed the viability and invasion potential of tongue cancer cells while promoting apoptosis and autophagy in vitro. Moreover, the synergistic inhibitory impact on the viability of cancer cells was notably observed with the combined use of *R. dentatus* L. extract and cisplatin.

While previous research has demonstrated the inhibitory effects of *R. dentatus* L. (leaves or roots) on various tumor cells, our study represents the first systematic analysis of the anti-tongue cancer effects of the phenolic aglycones of the aerial part of *R. dentatus* L. and underlying mechanisms of these bioactive compounds supported by network pharmacology.

The moderate cytotoxic activity of Rumex dentatus against cancer cells, combined with its low toxicity to normal cells, suggests the potential for developing plant-based therapies with reduced side effects compared to conventional chemotherapy. This observation provides a foundation for further pharmacological investigations into synergistic combinations of natural and synthetic agents. Additionally, it underscores the significance of elucidating the specific molecular pathways affected by natural compounds, thereby contributing to the theoretical understanding of cancer biology and apoptosis. The regulation of ATG7, p53, and BCL2 genes lends theoretical support to the concept that plant compounds can target multiple molecular pathways, offering a multifaceted approach to cancer therapy. The network pharmacology approach demonstrates the efficacy of bioinformatics in predicting the therapeutic potential of natural compounds. Furthermore, it emphasizes the complexity of cancer treatment, involving multiple signaling pathways and gene interactions. This evidence supports the hypothesis that natural compounds can be viable candidates for drug development, particularly when they exhibit favorable pharmacokinetic criteria such as appropriate absorption, distribution, metabolism, and excretion (ADME) properties.

## Data Availability

The datasets are available from the corresponding author upon reasonable request.

## Abbreviations

OSCC: Oral squamous cell carcinoma
HNO97: Tongue carcinoma cell line
KEGG: Kyoto Encyclopaedia of Genes and Genomes
EGFR: Epidermal growth factor receptor
UPLC-ESI–MS/MS: Ultra high-performance liquid chromatography-electrospray ionization-tandem mass spectrometry
DMEM: Dulbecco’s modified Eagle’s medium
OEC: Human Oral Epithelial Cell Line
TCA: Trichloroacetic acid
SRB: Sulforhodamine B
PI: Propidium iodide
FITC: Fluorescein isothiocyanate
RT-qPCR: Reverse transcription quantitative real-time PCR
OB: Oral bioavailability
DL: Drug-likeness
PPIs: Protein–protein interactions
GO: Gene ontology
BP: Biological process
MF: Molecular function
CI: Combination index
BC: Betweenness centrality
Cc: Closeness centrality
ROS: Reactive oxidative species
ECM: Extracellular matrix
